# Recovery of platinum group metals from spent automotive converters and their conversion into efficient recyclable nanocatalysts

**DOI:** 10.1007/s11356-022-24593-2

**Published:** 2022-12-15

**Authors:** Zuzanna Wiecka, Iuliana Cota, Bartosz Tylkowski, Magdalena Regel-Rosocka

**Affiliations:** 1 Poznan University of Technology, Institute of Chemical Technology and Engineering, ul. Berdychowo 4, 60-965 Poznań, Poland; 2 Eurecat, Chemical Technology Unit, Carrer de Marcel-lí Domingo, 43007 Tarragona, Spain

**Keywords:** Platinum group metals (PGM), Active nanoparticles, Titanium (IV) dioxide support, Recyclable catalysts, Spent automotive converters (SAC)

## Abstract

The study reported in this article has shown for the first time that strongly acidic solutions (pH < 0.5) obtained after hydrometallurgical treatment of spent automotive converters (SAC) may be valuable secondary sources of platinum group metal (PGM) nanoparticles (NPs). The PGM precipitation strongly depended on the solution pH; the yield of the precipitated PGM NPs increased considerably from 40% to almost 100% when the pH was adjusted to 7–8. To improve the NPs stability, commercial TiO_2_ was used as support to obtain efficient recyclable PGM@TiO_2_ catalysts. The size of the PGM NPs was smaller than 5 nm, while the diameter of the supported particles varied from 10 to 50 nm. The size and dispersion of PGM NPs on the support strongly depended on the pH of the medium: at pH < 0.5, the Pt and Pd NPs were significantly smaller than the NPs obtained at pH 7–8. Also, in the case of Pt@TiO_2_ and Rh@TiO_2_, the NPs were well dispersed on the support in contrast to the large agglomerates of Pd@TiO_2_. The PGM@TiO_2_ showed catalytic properties in the reduction of 4-nitrophenol to 4-aminophenol, particularly, at pH above 11. The highest conversion of 98% was obtained with 1% Pd@TiO_2_ at pH 14 after only 15 min. The catalyst was easily separated from the reaction mixture and reused in 7 consecutive cycles without significant loss of activity. The PGM@TiO_2_ synthesized from the real solution showed a similar catalytic activity (70% conversion at pH 14) as that obtained from model solution.

**Supplementary Information:**

The online version contains supplementary material available at 10.1007/s11356-022-24593-2.

## Introduction

Platinum group metals (PGM) are of great financial and industrial importance due to their applications in jewelry, electrical and electronic equipment, dental materials, and, most importantly, in automotive industry as indispensable components of catalytic converters (Johnson Matthey 2018, 2019). The demand for PGMs for industrial applications has increased significantly in the last decade due to the implementation of more restrictive emission legislations in several countries (Raymond and Sebrell 2019). Consequently, in the last five years, the price of PGMs has substantially increased: that of Pt—from 934 to 975 $/oz, that of Pd—from 798 to 2352 $/oz, and that of Rh was almost doubled, from 956 to 18,886 $/oz (Johnson Matthey 2021). To date, only about 25% of the global supply of PGM is covered by secondary resources (e.g., spent automotive converters (SAC) and jewelry), which means that the remaining 75% comes from primary resources located in a limited number of geographical areas where mining activities have a high environmental and societal impact (Kamunda et al. 2016; Kleinhenz 2017). As the supply of PGMs became precarious, especially in Europe (Communication from the Commission to the European Parliament 2017; Zientek et al. 2014) and due to their economic importance, PGMs have been classified as critical elements by the European Commission (Blengini et al. 2020). Furthermore, the contamination indexes, such as the risk assessment code, the contamination factor, and the global contamination factor, show that the SAC must be considered a hazardous waste (Bahaloo-Horeh and Mousavi 2020). Thus, effective recycling systems for PGM-containing secondary raw materials are of great importance, not only to eliminate hazardous materials from the environment but also to ensure a sustainable supply of the precious metals, while conserving the limited primary resources and maintaining a stable market price (Alonso et al. 2008; Bardi and Caporali 2014). Recycling of PGMs from SAC is a promising solution due to their high concentration in these materials (from hundreds to thousands ppm of PGM) compared to their abundance in primary ores (on average > 10 ppm PGM) (Hagelüken 2012).

On the one hand, pyrometallurgy has been widely applied for automotive catalyst recycling with promising PGMs recovery yields. Nevertheless, pyrometallurgical processes require special equipment, are energy-intensive, and generate large quantities of slag and environmental pollutants (SO_2_, NO_x_, CO, and dioxins). Consequently, extensive efforts have been made to develop energy-efficient and eco-friendly alternative processes to recover PGMs from SACs (Ding et al. 2019; Dong et al. 2015; Jha et al. 2013; Lee et al. 2020). On the other hand, hydrometallurgical methods such as leaching and liquid–liquid extraction (Paiva et al. 2017; Pośpiech 2012; Wiecka et al. 2022), roasting-assisted leaching (Trinh et al. 2019), oxidative leaching (Nogueira et al. 2014), or leaching with deep eutectic solvents (Lanaridi et al. 2022) have been proposed for PGMs recovery from secondary resources such as SACs, petrochemical catalysts, or electronic waste (Ding et al. 2019; Rzelewska-Piekut et al. 2021; Zheng et al. 2019).

Currently, global PGM production and recycling are based on hybrid pyro/hydro-metallurgical processes (i.e., BASF, Johnson Matthey, Umicore) (Dong et al. 2015), though hydrometallurgy is gaining growing attention due to lower processing temperatures, potential higher recovery rates, applicability on a smaller scale, safer handling of secondary streams (gaseous emissions vs. liquid effluents), etc. (Saguru et al. 2018). However, to be consistent with the requirements of the circular economy and sustainable development, it is important not only to recover PGMs efficiently from secondary sources but also to find a way to reuse them (Murthy and Ramakrishna 2022). Thus, using the PGMs solutions obtained from secondary sources as precursors to obtain catalytically active particles/nanoparticles of PGMs is of great interest.

The use of nanoparticles (NPs) as catalysts has several advantages over particles of a larger size (e.g., micrometric), including increased selectivity and activity of the material due to a larger number of active sites per unit area (Tahir et al. 2020). To obtain PGM NPs, different compounds can be used as metal precursors: (i) [PtCl_6_]^2−^, [PtCl_4_]^2−^, PtCl_2_, Pt(acac)_2_, [Pt(NH_3_)_4_](OH)_2_, and [Pt(NH_3_)_4_]Cl_2_ for Pt; (ii) PdCl_2_, [PdCl_4_]^2−^, Pd(NO_3_)_2_, and Pd(acac)_2_ for Pd; and (iii) RhCl_3_, RhCl_3_ $\bullet$ xH_2_O, and Rh_2_(TFA)_4_ for Rh (Kettemann et al. 2015; Selishchev et al. 2018; Xu et al. 2019; Jeyaraj et al. 2019). Depending on the solubility of the precursor salt and the type of the method employed, NPs can be precipitated in water or in an organic phase with the addition of a stabilizer.

Due to the small size and probable solubility in reaction media, it is not always easy to separate NPs from the solution; to overcome this problem, NPs are usually anchored onto a solid support. Several supports such as TiO_2_ (Shu et al. 2020; Yu et al. 2020), MgO (Hejral et al. 2013), Al_2_O_3_ (Benkhaled et al. 2006), SiO_2_ (Decarolis et al. 2018), and Fe_2_O_3_ (Amiri et al. 2019; Haruta et al. 1993) have been proposed to facilitate the NPs separation from the reaction medium. Furthermore, the use of a support prevents agglomeration of NPs, which are dispersed on the support surface, thus improving the catalytic properties of the material (Ndolomingo et al. 2020). The supported PGMs have been described in literature as catalysts for a variety of reactions, including selective hydrogenation reactions, CO oxidation, propane dehydrogenation, and photocatalytic H_2_ evolution reaction (Liu et al. 2019; Martínez-Castro et al. 2020; Repousi et al. 2017; Wu et al. 2016).

The present article describes the synthesis of PGM NPs deposited on TiO_2_ from model and real solutions obtained by leaching of SAC purified by liquid–liquid extraction and stripping. The catalytic properties of the obtained materials were determined in the reduction of 4-nitrophenol (4-NP) to 4-aminophenol (4-AP) as a model reaction.

## Experimental

### Materials and methods

One-component model solutions of compositions similar to that of real leach solutions were prepared by dissolving the required amounts of PtCl_4_ (94%, Sigma Aldrich), PdCl_2_ (99.9%, Sigma Aldrich), or RhCl_3_ (99.9%, Sigma Aldrich) in acidic medium. Polyvinylpyrrolidone (PVP) (Mw – 40,000, Sigma Aldrich) and sodium borohydride (NaBH_4_) (> 99.0%, Acros) were used as a stabilizing agent and a reducer, respectively. A portion of 3 M HNO_3_ (60%, Acros) was used for Pt, 0.5 M HCl (pure p.a., Chempur, Poland) for Pd, and for Rh the acid mixture was twice diluted with HCl/H_2_SO_4_/H_2_O_2_ (HCl and H_2_SO_4_ – pure p.a., Chempur, Poland, H_2_O_2_ – ACS reagent, Avantor, Poland). A 5 M NaOH solution (pure p.a., POCH, Poland) was used to adjust the pH during precipitation of Pd and Rh. Commercial TiO_2_ (Aeroxide® TiO_2_ P25) was used as a support for the preparation of supported PGM catalysts. Stripping solutions obtained according to the method previously reported for the recovery of precious metals from SACs were used as real PGM precursor solutions (Wiecka et al. 2022).

### Instruments

Metal ion concentrations in the aqueous samples were measured using an atomic absorption spectrometer (AAS) (ContrAA 300, Analytik Jena) at 266.0, 244.8, and 343.5 nm wavelengths for Pt(IV), Pd(II), and Rh(III), respectively. Transmission electron microscopy (TEM) images were recorded in a JEOL 1011 instrument with an accelerating voltage of 100 kV (TEM). Scanning electron microscopy and energy dispersive X-ray spectroscopy (SEM–EDS, SEM FEI Quanta 250 FEG) and an atomic force microscope (AFM, NX10, Park Systems) were used to characterize the synthesized material. Absorption spectra were recorded on a spectrophotometer (Specord 40, Analytik Jena) equipped with a quartz cuvette. The surface area (SBET) was calculated according to the Brunauer- Emmett-Teller model (BET, Micromeritics ASAP 2420) method. The samples were degassed at vacuum at 120 °C for 24 h. The total pore volume (V) was determined from the amount of vapor adsorbed at *P*/*P*_0_ = 0.99 and average pore diameter was determined assuming a cylindrical shape using 4 V/SBET formula.

### Synthesis of PGM nanoparticles supported on TiO_2_

Catalytic materials containing 0.1, 0.5, and 1 wt.% of PGM in relation to the amount of TiO_2_ support were prepared. TiO_2_ was chosen as a support due to its high specific surface area and strong interaction with metal nanoparticles, which improved the catalytic stability and activity of the supported NPs. Moreover, TiO_2_ has good mechanical resistance and stability in acidic and oxidative environments.

The precipitation reaction yield (E_p_) was determined as follows:

1 $${E}_{p}=\frac{\left({m}_{0}-{m}_{1}\right)}{{m}_{0}}\bullet 100\mathrm{\%}$$

where *m*_0_ and *m*_1_ are the masses of PGM ions in the solutions before and after precipitation, respectively. The mass variation of the PGMs in the solution before and after precipitation was estimated using AAS measurements. During the synthesis of 0.1, 0.5, and 1% PGM@TiO_2_, the pH of the solution was adjusted to 7–8. For comparison purpose, 1% PGM@TiO_2_ material was also synthesized without pH adjustment (pH < 0.5).

### Synthesis of Pt@TiO_2_ from model solutions

A portion of 2.5 ml of PVP solution was added to 15 ml of Pt(IV) precursor in 3 M HNO_3_ and stirred for 10 min. Then, 2.5 ml of NaBH_4_ solution was added dropwise to the mixture upon stirring for 10 min. The molar ratio of the Pt precursor, the reducing agent, and the stabilizing agent was 1:2:1. The pH was adjusted to 7–8 using 5 M NaOH solution. Afterwards, 0.5 g of TiO_2_ support was added and the mixture was stirred for 2 h. The resulting precipitate was centrifuged (5 min at 9000 rpm) and washed twice with ethanol and twice with MiliQ water. The materials were then dried in the oven at 50 °C for 2 days. These materials were labeled as Pt@TiO_2_.

### Synthesis of Pd@TiO_2_ from model solutions

A portion of 2.5 ml of PVP solution was added to 15 ml of Pd(II) precursor in 0.5 M HCl and stirred for 10 min. Further, the same steps as for the synthesis of Pt@TiO_2_ were followed. These materials were labeled as Pd@TiO_2_.

### Synthesis of Rh@TiO_2_ from model solutions

A portion of 2.5 ml of PVP solution was added to 15 ml of Rh(III) precursor in a mixture of 5.5 M HCl, 9 M H_2_SO_4_, and 6.4 M H_2_O_2_ (volume ratio: 0.9:0.05:0.05). Since Rh did not precipitate when 2.5 ml of NaBH_4_ solution was used, an additional 10 ml of NaBH_4_ solution was added dropwise and the mixture was stirred for 10 min. The molar ratio of the Rh precursor, the reducing agent, and the stabilizing agent was 1:8:1. Further, the same steps as for the synthesis of Pt@TiO_2_ were followed. These materials were labeled as Rh@TiO_2_.

### Synthesis of Pt@TiO_2_ from real solution

The procedure of PGM recovery from spent automotive converters was fully described in a previous publication of our group (Wiecka et al. 2022). Concentrations of metals in the solution after leaching, extraction, stripping, and precipitation are shown in the Supplementary Information, Table A1. Briefly, 2.5 ml of PVP solution was added to 15 ml of Pt(IV) precursor in 3 M HNO_3_ and stirred for 10 min. Then, 2.5 ml of NaBH_4_ solution was added dropwise to the mixture for 10 min upon stirring. The molar ratio of the Pt precursor, the reducing agent, and the stabilizing solution was 1:2:1. A 5 M NaOH solution was used to adjust the pH to 7–8. Afterwards, 0.37 g of TiO_2_ support was added and the mixture was stirred for 2 h. Since the solution volume obtained after the precipitation from the real leach solution was different from the one obtained from the model solution, the quantity of the support used was adjusted according to these conditions. The resulting precipitate was centrifuged (5 min at 9000 rpm) and washed twice with ethanol and twice with MiliQ water.

### Catalytic reduction of 4-nitrophenol to 4-aminophenol

To test the catalytic performance of the obtained materials, the reduction of 4-nitrophenol (4-NP) (pKa 7.15) to 4-aminophenol (4-AP) (pKa 5.48 and 10.46 for the amine and hydroxy functional groups, respectively) with NaBH_4_ as hydrogen donor in aqueous medium was carried out (Website Pubchem 2022). To keep 4-NP in anionic form in the solution, the pH of the initial reaction solution was increased to 11 by adding NaOH. In a typical reaction, 1.5 ml of 15 mM NaBH_4_ was added dropwise to a reaction mixture containing 3.5 ml of 0.05 mM 4-NP solution and 6 mg of Pd@TiO_2_, Pt@TiO_2_ or Rh@TiO_2_ catalyst. The reaction progress was monitored by UV–vis spectroscopy at a fixed wavelength (400 nm = λ_max_ of 4-NP) for samples taken every 5, 15, and 30 min.

To study the recyclability of the catalyst, the most effective catalysts synthesized from either the model solution or the real solution were recovered and reused in the 4-NP reduction reaction for several cycles. In the first cycle, 6 mg of catalyst was used, which were recovered at the end of the reaction by centrifugation (10 min at 5000 rpm) and washed twice with MiliQ water before use in the next cycle.

## Results

### Effect of pH on the efficiency of PGM-NP precipitation

The most important parameters for the synthesis of PGM NPs are the type of method employed (e.g., chemical reduction), the reducing agent used, the pH of the reaction mixture, temperature, the addition of a stabilizing agent, and the reaction time (Jeyaraj et al. 2019; Patra and Baek 2014). The selection of the appropriate parameters should lead to the formation of stable NPs that can be used as catalysts.

The pH of the metal precursor solution is of great importance during the precipitation step because it directly influences the degree of the precursor hydrolysis (Mäki-Arvela and Murzin 2013). Thus, at different pH values, the metal precursor particles can have different sizes, e.g., chloride ligand is replaced by a hydroxyl ligand when increasing the pH. Moreover, in the case of supported NPs, the pH can influence the location of the precipitated particles on the surface or inside the pores of the support, depending on the type of interactions that take place between the support and the metal precursor particles.

**Fig. 1:**
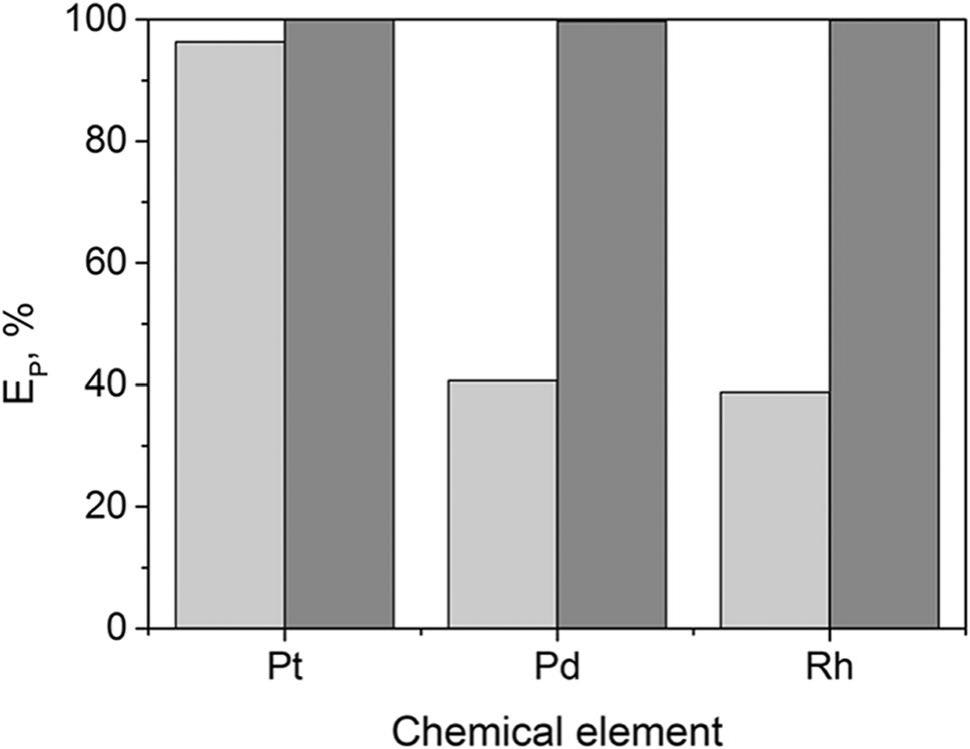
E_P_ of Pt(IV), Pd(II), and Rh(III) without pH adjustment (  ) and at pH 7–8 (  )

Considering the influence of pH on the yield of precipitated PGM NPs, in the present study, the precipitation step was performed without pH adjustment in an acidic medium (pH < 0.5) and with pH adjustment (pH 7–8) followed by the addition of a reducing agent (Fig. 1).

The results (Fig. 1) showed that the change in pH did not significantly affect the yield of precipitated Pt; however, considerable differences were observed for Pd and Rh. In both cases, at pH < 0.5, *E*_*P*_ did not exceed 40%, but when increasing pH to 7–8, the *E*_*P*_ values were close to 100%. During the synthesis of Pd and Rh NPs without pH adjustment, it was noted that the solution changed from colorless to brown over time, suggesting that the material synthesized was dissolving. To confirm whether the obtained Pd NPs dissolved over time, samples of the precipitation medium were taken at 0.5, 1, 3, and 24 h and analyzed by AAS. It is known that under acidic conditions (pH < 0.5), the precursor Pd(II) exists in the form of tetrachlorocomplex [PdCl_4_]^2−^ with a maximum of absorbance at 222 and 278 nm (Zhao et al. 2017). Thus, a comparison of UV–vis spectra of the solution after Pd precipitation at different times (0.5 to 24 h) showed that after 24 h, the Pd NPs yield decreased to 20% (Supplementary Information, Fig. A1), indicating a partial dissolution of the NPs in an acidic environment (pH < 0.5).

Therefore, to prevent the dissolution of the metal NPs in the acidic solutions, the pH during the precipitation step was increased to 7–8 in further experiments.

### Characterization of the materials obtained from model solutions

The particle size distribution of TiO_2_ support and the PGM@TiO_2_ NPs was determined by AFM. The results showed that TiO_2_ particle diameters were below 40 nm (Supplementary Information, Fig. A2) before and after supporting the PGM NPs indicating that the TiO_2_ support was stable during the deposition process.

**Fig. 2:**
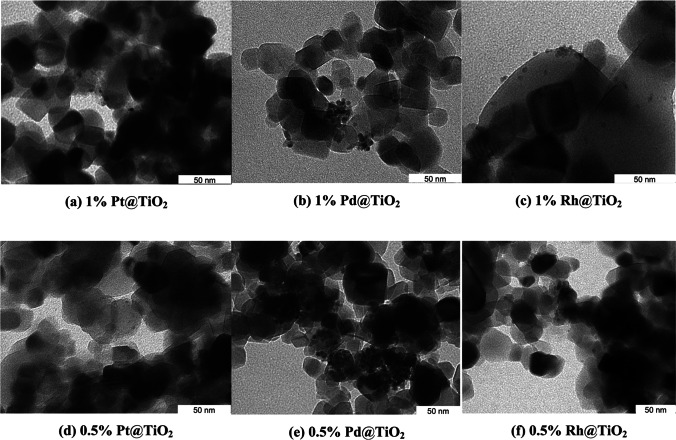
TEM images of samples with 1 wt% PGM (**a**, **b**, and **c**) and 0.5 wt% PGM (**d**, **e**, and **f**) on TiO_2_ support

Furthermore, to confirm PGM NP deposition on the TiO_2_, a TEM analysis was performed (Fig. 2). TEM results of the PGM@TiO_2_ synthesized without pH adjustment (Supplementary Information, Fig. A3) and with pH adjustment (Fig. 2) showed that TiO_2_ particles have a similar structure, indicating that pH does not affect the morphology or size of TiO_2_ support. It was also observed that after supporting the PGM NPs, the TiO_2_ particles agglomerated and formed complex structures. In the case of 1% Rh@TiO_2_, small aggregates were detected, while for 1% Pd@TiO_2_, large agglomerates with a diameter up to 600 nm were observed (Fig. 2). TEM images were not taken for samples containing 0.1 wt.% PGM due to the low concentration of the metal.

The diameter of the PGM NPs measured from the TEM images did not exceed 5 nm (Table 1) in all samples. The size of Pt and Pd particles synthesized without pH adjustment (pH < 0.5) was significantly smaller than that of the particles synthesized at pH 7–8 (3.5 to 4.5 mm), while for Rh, the decrease in particle diameter was not significant. Similar results have been obtained in the study of Cheng et al. (2018), when Pd was synthesized on SiO_2_ support using solutions of various HCl concentrations (from 0.1 to 5 M). It has been observed that high concentrations of H^+^ and Cl^−^ affected the dispersion, size, and distribution of Pd nanoparticles on SiO_2_. When the HCl concentration was increased from 0.1 to 2 M, the size of the Pd particle dropped dramatically from 24.2 to 5.6 nm due to the stronger electrostatic interactions between the metal and the support. In the samples Pt@TiO_2_ (Fig. 2a and d) and Rh@TiO_2_ (Fig. 2c and f), the Pt and Rh NPs were well dispersed on the TiO_2_ support while, in contrast, large agglomerates of these NPs were visible for Pd@TiO_2_ (Fig. 2b and e). A similar phenomenon has been reported for the PGM@TiO_2_ samples obtained by the sol–gel, wet-impregnation, and hydrothermal methods (García-Zaleta et al. 2016; Macino et al. 2019; Yu et al. 2020). The García-Zaleta team proposed the synthesis of Pt or Pd NPs on a TiO_2_ support by the sol–gel method using stoichiometric amounts of various precursors in an acidic environment. Subsequently, the obtained catalysts were thermally treated at 500 °C and milled to obtain particles of similar size. Depending on the molar ratio of the metal used, the obtained Pt-NPs were of about 12 nm, while the Pd-NPs showed a tendency to form agglomerates of a size from 15 to even 400 nm (García-Zaleta et al. 2016). The agglomeration mechanism is associated with the thermodynamic instability of NPs, excess surface energy, solution pH, and ionic strength (Martínez-Abad 2011; Singer et al. 2019). Thus, the degree of agglomeration depends on stage of nucleation and growth, which determines the size and morphology of the obtained NPs. During precipitation of NPs in the nucleation step, numerous small crystals are formed, which are able to form more thermodynamically solid particles, resulting in agglomerates formation. Small metal particles have a high surface energy; therefore, they are more susceptible to agglomeration due to thermodynamics (Cushing et al. 2004; Mäki-Arvela and Murzin 2013)

**Table 1:** The size of the PGM NPs deposited on the TiO_2_ support (diameter measured with TEM)

Content of PGM	Diameter of PGM, nm		
	Pt(IV)	Pd(II)	Rh(III)
pH 7–8			
1%	4	4.5	3.5
0.5%	4	4.5	3.5
Without pH adjustment (pH < 0.5)			
1%	< 1.5	< 1.5	3

**Fig. 3:**
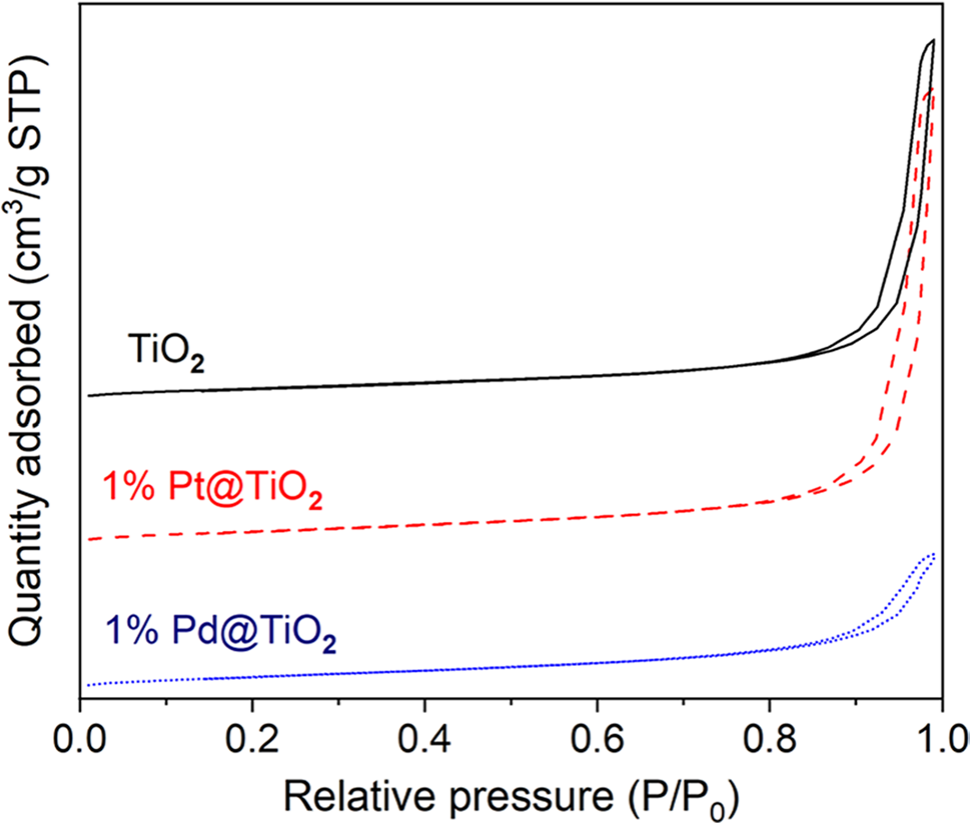
N_2_ isothermal adsorption–desorption as-prepared TiO_2_, 1% Pt@TiO_2_, and 1% Pd@TiO_2_

The nitrogen adsorption–desorption isotherms corresponding to the support TiO_2_, 1% Pt@TiO_2_, and 1% Pd@TiO_2_ materials (Fig. 3) are type IV (according to IUPAC classification) indicating that the mesoporous structure of TiO_2_ was maintained after Pt and Pd NP deposition. The isotherms of this type indicate a weak adsorption at low relative pressure and a H1-type hysteresis loop at higher relative pressure (*P*/*P*_0_ = 0.80–0.90) suggesting that the material is mesoporous with well-defined cylindrical pore channels.

The Brunauer–Emmett–Teller (BET) surface area, average pore diameter, and total pore volume of the samples are presented in Table 2.

**Table 2:** Structural properties of TiO_2_ support and supported 1% Pt@TiO_2_ and 1% Pd@TiO_2_ materials

Material	BET surface area (m^2^/g)	BJT pore volume (cm^3^/g)	BJT pore diameter (nm)	Micropore area (m^2^/g)
TiO_2_	53.40	0.15	11.20	2.268
1% Pt@TiO_2_	43.96	0.38	34.67	*
1% Pd@TiO_2_	52.22	0.49	33.89	1.724

^*^The micropore area is not reported because either the micropore volume is negative or the calculated external surface area is larger than the total surface area

Commercial TiO_2_ used as support for PGM NPs has a BET surface area of 53.4 m^2^/g, typical of mesoporous TiO_2_ (~ 49.8 m^2^/g). After Pd deposition, the area slightly decreased to 52.22 m^2^/g, while in the case of Pt deposition, the decrease was more significant, to 43.96 m^2^/g. The loss of surface area after PGM NP deposition can be correlated with the closure of micropores within TiO_2_, as revealed in the N_2_ isotherm measurement. A significant decrease of 1% Pt@TiO_2_ surface area is in agreement with the disappearance of microporosity of TiO_2_ after Pt deposition.

### Catalytic properties of PGM@TiO_2_ obtained from model solutions

All catalysts tested were prepared with pH adjustment (pH 7–8) during the precipitation step to increase the precipitation yield and to prevent dissolution of the obtained NPs. Reduction of 4-NP to 4-AP was carried out in order to check the catalytic activity of the obtained PGM@TiO_2_, which was estimated from the degree of 4-NP conversion. For the sake of comparison, the reaction with TiO_2_ support alone was carried out, which confirmed the lack of activity of the support itself (results not shown). The reactions were carried out at pH 11 in order to maintain 4-NP in anionic form in the solution. All reactions were performed in triplicate, and the calculated error in each experiment did not exceed 10%.

For 0.1% PGM@TiO_2_ tested, the 4-NP conversion was low, i.e., 6.4, 10.9, and 40.4% for Pt@TiO_2_, Pd@TiO_2_, and Rh@TiO_2_, respectively. These results confirmed that 0.1% PGM content in the PGM@TiO_2_ material was too low to ensure efficient performance. The highest 4-NP conversion was obtained with 1% Pd@TiO_2_ followed by 0.5% Pd@TiO_2_ and 0.5% Rh@TiO_2_, reaching 98.2, 95.7, and 73.4%, respectively (Supplementary Information, Table A2). These results indicate that Pd@TiO_2_ has a higher catalytic activity than Pt@TiO_2_, probably due to the increased surface area (52.22 m^2^/g) of the former compared to the latter (43.96 m^2^/g). The decrease in the catalytic activity can be correlated with the higher surface area of Pd@TiO_2_, which is in agreement with the observations made by Grzeschik et al. (2020). A significantly lower conversion obtained with 1% Rh@TiO_2_ compared to that obtained with the use of 0.5% Rh@TiO_2_ may be due to the aggregation of Rh NPs, as observed in Fig. 2c, and thus, the limitation of the active sites availability. A similar decrease in the catalytic activity of Rh@CeO_2_ with increasing rhodium loading, attributed to the aggregation of Rh NPs, has been observed previously by Akbayrak et al. (2016).

The influence of reaction time on the 4-NP reduction was investigated using 1% PGM@TiO_2_ catalysts (Supplementary Information, Fig. A4). As expected, the concentration of 4-NP decreased with time; in the reaction catalyzed by 1% Pd@TiO_2_ after only 5 min the 4-NP conversion increased by 90% and after 15 min reached 95%. The end of the reaction was also confirmed by the change in the color of the reaction medium from yellow to colorless. The reactions catalyzed by 1% Pt@TiO_2_ and 1% Rh@TiO_2_ were slower. After 30 min, the 4-NP conversion increased to 13% in the presence of 1% Pt@TiO_2_ and 44% in the presence of 1% Rh@TiO_2_, and after this time, the dependence reached a plateau. Some experiments were conducted for 40 min but the degree of 4-NP conversion did not change significantly. Based on these results, the reaction time for the following experiments was set at 30 min.

**Fig. 4:**
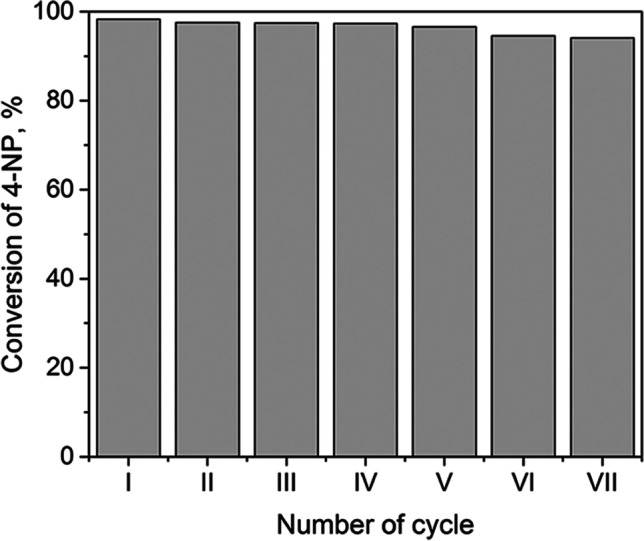
4-NP conversion at pH 11 after several cycles with 1% Pd@TiO_2_ as catalyst

The most efficient catalyst, 1% Pd@TiO_2_, was selected for the study of the catalyst reusability in several cycles of 4-NP reduction at pH 11. The sample Pd@TiO_2_ showed excellent reusability as 4-NP conversion did not change significantly after 7 consecutive cycles (Fig. 4). A small decrease in its activity after each cycle could be due to the loss of catalyst during separation from the reaction mixture.

In the study by Grzeschik et al. (2020), it was observed that the pH value of the reaction medium played a critical role in the kinetics of 4-NP reduction to 4-AP, as both the rate constant and the reaction order were strongly influenced by pH. This behavior was attributed to the pH-dependent hydrolysis of the reducing agent NaBH_4_ in water. In each hydrolysis step, molecular hydrogen was produced as a second reducing agent, and two competing pathways were identified: slow hydride versus fast hydrogen reduction. At pH ≥ 13, a predominantly hydride-induced reaction occurs (first order reaction), while at pH ≤ 10, a mostly hydrogen-driven reaction with a fractional reaction order lower than 1 is observed.

**Fig. 5:**
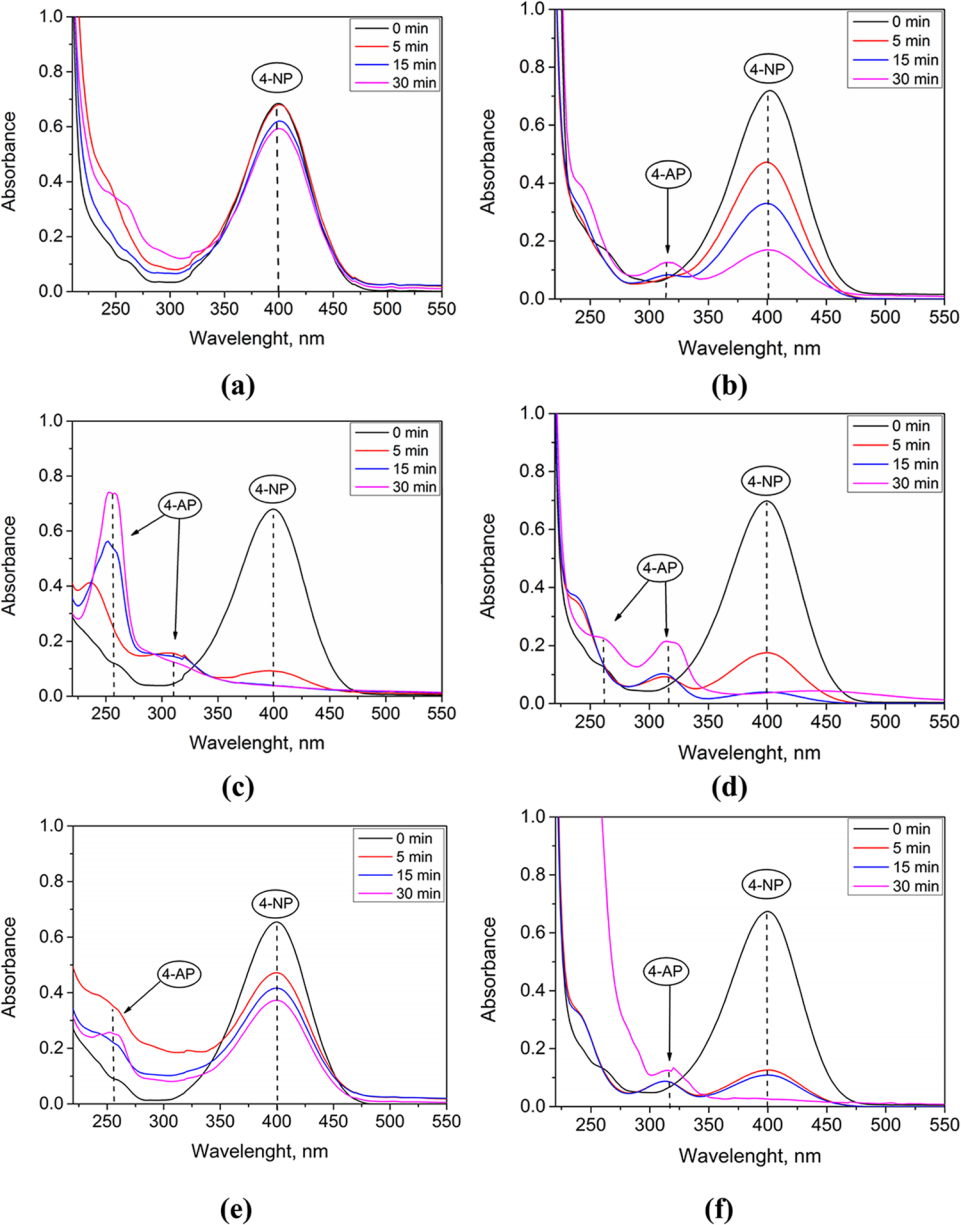
UV–vis spectra of 4-NP reaction medium at different reduction times in the presence of 1% Pt@TiO_2_ (**a**-**b**), Pd@TiO_2_ (**c**-**d**), and Rh@TiO_2_ (**e**–**f**) at pH 11 (**a**, **c**, and **e**) and 14 (**b**, **d**, and **f**). The catalyst mass: 6 mg

To check if the supported PGMs@TiO_2_ from our study exhibits the same behavior as the unsupported PGM NPs from the Grzeschik’s study, the reduction of 4-NP with 1% PGM@TiO_2_ was performed at pH 14 in triplicate. The calculated error for each reaction was less than 10%. The UV–vis spectra of the 4-NP solution obtained at different times of the reduction reaction at pH 11 or 14 are presented in Fig. 5.

In the reaction catalyzed by Pd@TiO_2_, the increase in pH from 11 to 14 did not significantly affect the conversion of 4-NP; in both cases, the conversion was around 98%. However, changing the pH of the reaction made a significant difference in the catalytic activities of 1% Pt@TiO_2_ and Rh@TiO_2_. In the presence of 1% Pt@TiO_2_, after 30 min, the 4-NP conversion was 13% at pH 11, while at pH 14, it was around 80%. For the reaction catalyzed by 1% Rh@TiO_2_ after 30 min, the 4-NP conversion was 45% compared to 90% at pH 14 (Supplementary Information, Table A3).

In the spectrum of the product of the reaction performed at pH 14 in the presence of all three catalysts, besides the shoulder at 260 nm attributed to 4-AP, a new and intense peak corresponding also to 4-AP appeared at 320 nm, indicating that in strongly basic solutions (pH 14), the concentration of aminophenolic ion (pK_2_ 10.46) was higher than at pH 11 (Website Pubchem 2021). As observed by Grzeschik et al. (2020), at pH 14 the efficiency of 4-NP reduction increased in the presence of all the catalysts tested due to the change in the mechanism of reduction with NaBH_4_, which explains the difference in the 4-NP conversion when using 1% Pt@TiO_2_ and 1% Rh@TiO_2_ at pH 11 and 14.

### Characterization of Pt@TiO_2_ obtained from real solutions

After examination of the model solutions, precipitation of Pt@TiO_2_ was carried out from the real solutions. To the best of our knowledge, this is the first time that PGM NP formation from real solutions after leaching of SACs is described. The difficulty in NP formation from the real solutions lies in the high acidity of these solutions (pH < 0.5) and the presence of accompanying/contaminating ions of non-precious metals. Therefore, some additional hydrometallurgical steps presented in a previous study of our group (Wiecka et al. 2022) are necessary to prepare the feed for NP precipitation.

The precious metals were derived from the Pt–Rh spent catalyst provided by a Polish company. The composition of the solution (after stripping, pH < 0.5) used for the precipitation is shown in Supplementary Information, Table A1. The precipitation yield of Pt NPs on TiO_2_ support from the real solution was 99%. The SEM–EDS spectrum (Supplementary Information, Fig. A5) confirmed that small amounts of Fe, Cu, and Zn present in the stripping solution were deposited on TiO_2_ together with Pt. SEM images, presented in Supplementary Information, Fig. A6, showed that Pt NPs were well dispersed on the TiO_2_ support.

**Fig. 6:**
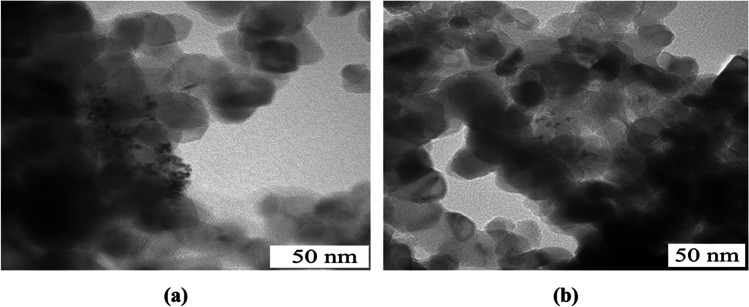
TEM images of samples with 1 wt% Pt on TiO_2_ support from real solution

The particle size distribution determined by AFM analysis showed the presence of TiO_2_ single particles of the sizes in the range of 10 to 50 nm and also small agglomerates (Supplementary Information, Fig. A7). In addition, TEM images allowed estimation of the size of metal nanoparticles to be below 5 nm. Images taken at various points (a) and (b) showed both the location of agglomerates and well-dispersed particles (Fig. 6).

### Catalytic properties of Pt@TiO_2_ obtained from real solutions

The catalytic properties of Pt@TiO_2_ synthesized from the real solution were determined in the 4-NP reduction at pH 11 or 14 (Supplementary Information, Fig. A8). The reactions were performed in triplicate and the calculated error for each reaction was less than 7%. The catalytic activities of Pt@TiO_2_ obtained from the real and model solutions were similar with a 4-NP conversion around 16% at pH 11 indicating that the metal impurities deposited on the TiO_2_ support together with Pt NPs did not have any influence on the catalytic activity of the Pt@TiO_2_ material. However, when the reaction pH was increased to 14, the 4-NP conversion significantly increased to 75.5%. In order to check the stability and reusability of the catalyst synthesized from the real solution, several consecutive 4-NP reduction reactions at pH 14 were carried out with the recovered catalyst.

**Fig. 7:**
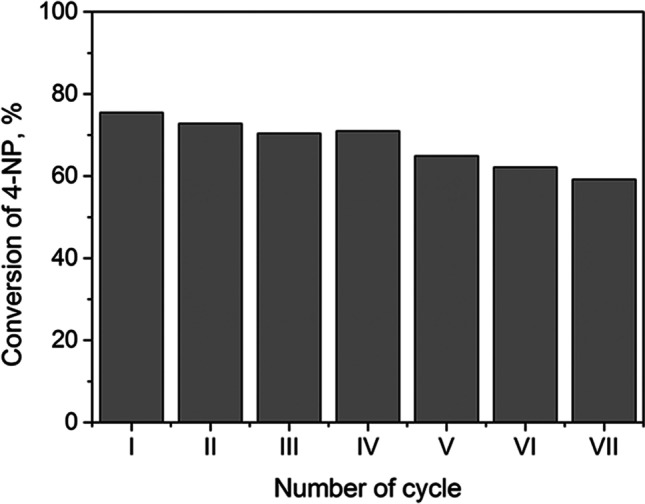
Degree of 4-NP conversion after several reactions with 1% Pt@TiO_2_ obtained from the real solution

The conversion of 4-NP in the first cycle reached 76% and slowly decreased after each cycle down to 59% after the 7^th^ cycle (Fig. 7). The advantageous feature of PGM@TiO_2_ materials is that they can be easily separated from the reaction mixture and reused for at least 7 cycles without a significant loss of activity. Therefore, future research should focus on improving the stability of these nanocatalysts (PGM@TiO_2_) synthesized from the solution obtained after leaching of spent automotive converters. Furthermore, also a variety of catalytic reactions should be investigated, in which PGM@TiO_2_ could participate as a catalyst, e.g., hydrogenation of 3-nitrostyrene, methanol oxidation, formaldehyde oxidation reactions, propane reforming (Macino et al. 2019; Antolini 2018; Wang et al. 2021; Yu et al. 2018), or environmental degradation of some micropollutants, also known as “contaminants of emerging concern,” present on a watch list of substances for monitoring in water, set out by EU in Directive 2008/105/EC.

## Conclusion

It has been demonstrated for the first time that strongly acidic solutions (pH < 0.5) obtained after hydrometallurgical treatment of spent automotive converters (after pH correction) can be a valuable source of precious metals in the form of catalytically active nanoparticles on TiO_2_ support, provided that the solution is neutralized beforehand.

The results obtained for model solutions showed that the pH of the solution had a significant influence on the precipitation process: *E*_*P*_ of Pd and Rh in acidic medium (pH < 0.5) did not exceed 60%, while at pH 7–8, *E*_*P*_ values of Pt, Pd, and Rh were close to 100%. TEM results confirmed the deposition of PGM NPs of diameters below 5 nm on the TiO_2_ support. Pt and Rh NPs were well dispersed on the TiO_2_ support, while large and extensive agglomerates were visible for Pd@TiO_2_.

All PGM@TiO_2_ were catalytically active in the reduction of 4-nitrophenol to 4-aminophenol in an alkaline environment. Furthermore, an increase in pH from 11 to 14 seemed to greatly influence the 4-nitrophenol conversion in the presence of Pt@TiO_2_ or Rh@TiO_2_ catalysts, due to a change in the reduction mechanism that follows the boron hydride induced pathway. The most efficient catalyst was 1% Pd@TiO_2_ which ensured a 4-nitrophenol conversion of 95% after only 15 min. Moreover, the catalyst was easily separated from the reaction mixture and could be reused in 7 consecutive cycles without significant loss of activity. It is worth mentioning that the catalytic activities of Pt@TiO_2_ obtained from model and real solutions were similar.

The supported PGM@TiO_2_ catalysts showed promising catalytic potential, which opens an important pathway for the recovery and recycling of PGMs from secondary resources in accordance with the requirements of the circular economy and sustainable development.

## Supplementary Information

Below is the link to the electronic supplementary material.

Supplementary file1 (DOCX 17.8 MB)

## Data Availability

The data presented in this study are available on request from the corresponding authors.
