# Supplementary material for: Recovery of platinum group metals from spent automotive converters and their conversion into efficient recyclable nanocatalysts
Source: Environ Sci Pollut Res Int. 2022 Dec 15;30(39):90168–79. doi: 10.1007/s11356-022-24593-2 (PMC10439850; doi:10.1007/s11356-022-24593-2)
Supplement: Supplementary file 1 — Supplementary file1 (DOCX 17.8 MB) [file 11356_2022_24593_MOESM1_ESM.docx]

**Supplementary Information to
Recovery of platinum group metals from spent automotive converters and their conversion into efficient recyclable nanocatalysts**

**Zuzanna Wiecka^1^, Iuliana Cota^2^, Bartosz Tylkowski^2^, Magdalena Regel-Rosocka*^1^**

^1^ Poznan University of Technology, Institute of Chemical Technology and Engineering, ul. Berdychowo 4, 60-965 Poznań, Poland

^2^ Eurecat, Chemical Technology Unit, Carrer de Marcel-lí Domingo, 43007 Tarragona, Spain

* Corresponding author:

Magdalena Regel-Rosocka [magdalena.regel-rosocka@put.poznan.pl](mailto:magdalena.regel-rosocka@put.poznan.pl)

Concentrations of metal ions in the solution after leaching (feed solution) of spent automotive converter with HCl/H_2_O_2_/H_2_SO_4_ mixture are shown in Table A1. This solution was used as feed for further hydrometallurgical treatment to separate and purify platinum group metals. The successful separation of Pt(IV) from the feed is clearly visible in the stripping solution. The concentration values after precipitation confirm almost complete removal of Pt(IV) from the aqueous solution in the form of Pt deposition.

Table A2 presents conversion of 4-NP to 4-AP obtained in the contact with catalytically active PGM NPs.

**Table A1** Metal ion concentrations after leaching with HCl/H_2_O_2_/H_2_SO_4_ (S/L = 1/100 g/cm^3^, 3 h, 70 °C, 300 rpm), extraction (organic phase: 0.005 M Cyphos IL 101, A/O = 6), stripping (3 M HNO_3_, O/A=2) and after precipitation

| **Chemical element** | **Concentration of metal ions, mg/dm^3^** | | | |
| --- | --- | --- | --- | --- |
|  | **Feed solution** | **Extraction** | **Stripping** | **After precipitation** |
| Pt(IV) | 22.79 | 1.37 | 247.08 | 1.20 |
| Rh(III) | 2.03 | 2.03 | 0 | 0 |
| Fe ions | 2.27 | 0 | 30.11 | 0.03 |
| Mg(II) | 24.87 | 23.34 | 0.64 | 0.26 |
| Cu(II) | 0.14 | 0.12 | 0.63 | 0 |
| Zn(II) | 0.67 | 0.21 | 6.93 | 0 |

**Table A2** Average values of 4-NP conversion obtained in the presence of 0.1, 0.5 or 1% PGM@TiO_2_ catalysts

| **Content of PGM, wt%** | **Conversion_4-NP_, %** | | | |
| --- | --- | --- | --- | --- |
|  | **Pt**@TiO_2_ | **Pd**@TiO_2_ | **Rh**@TiO_2_ |  |
|  | **pH 11** | | | |
| 1.0 | 13.3 | 98.2 | 44.0 | |
| 0.5 | 17.0 | 95.7 | 73.4 | |
| 0.1 | 6.40 | 10.9 | 40.4 | |

Reaction conditions: 6 mg catalyst, 30 min reaction time, ambient temperature, reaction medium pH 11.

Average 4-NP conversion values obtained in the presence of 1% PGM@TiO_2_ catalysts over time and at different pH are shown in Table A3.

**Table A3** Average values of 4-NP conversion obtained in the presence of 1% PGM@TiO_2_ catalysts during the reaction time

| **Time, min** | **Conversion_4-NP_, %** | | | |
| --- | --- | --- | --- | --- |
|  | **Pt**@TiO_2_ | **Pd**@TiO_2_ | **Rh**@TiO_2_ |  |
|  | **pH 11/14** | | | |
| 5 | 0.7/35.5 | 87.6/77.8 | 28.3/84.7 | |
| 15 | 9.5/56.1 | 95.5/98/1 | 36.8/87.3 | |
| 30 | 13.3/81.1 | 98.2/98.5 | 44.0/92.1 | |

Reaction conditions: 6 mg catalyst, ambient temperature

Fig. A1 shows the UV-vis spectra of the solution during Pd NP precipitation registered over time to determine which form of Pd was present in the solution. The comparison of the UV-vis spectra over time shows that the absorbance increases from 0.5 to 24 h, while the precipitation yield values after 0.5, 1, 3 and 24 h of the reaction decrease and finally stabilize at about 20%.

| 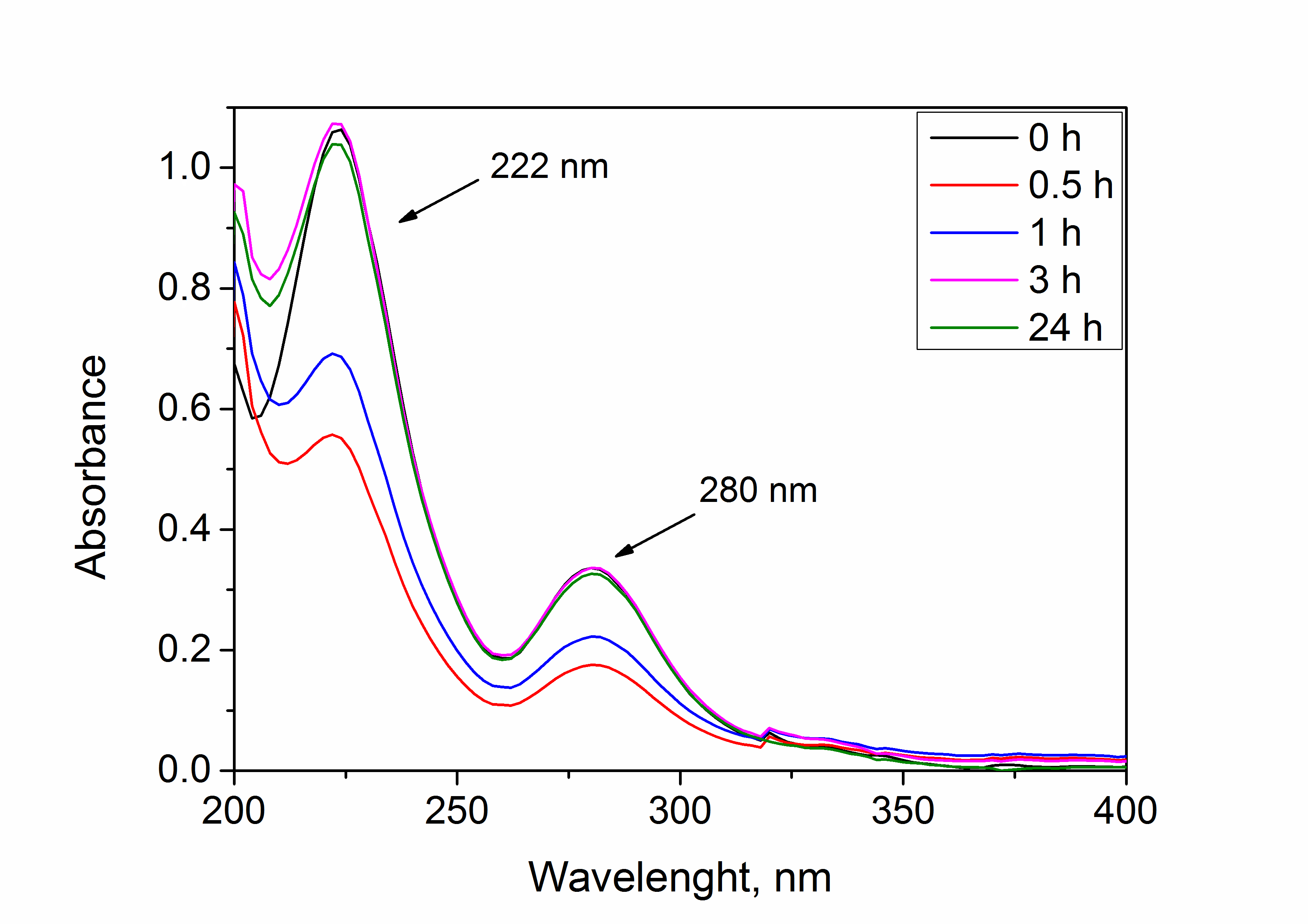 | 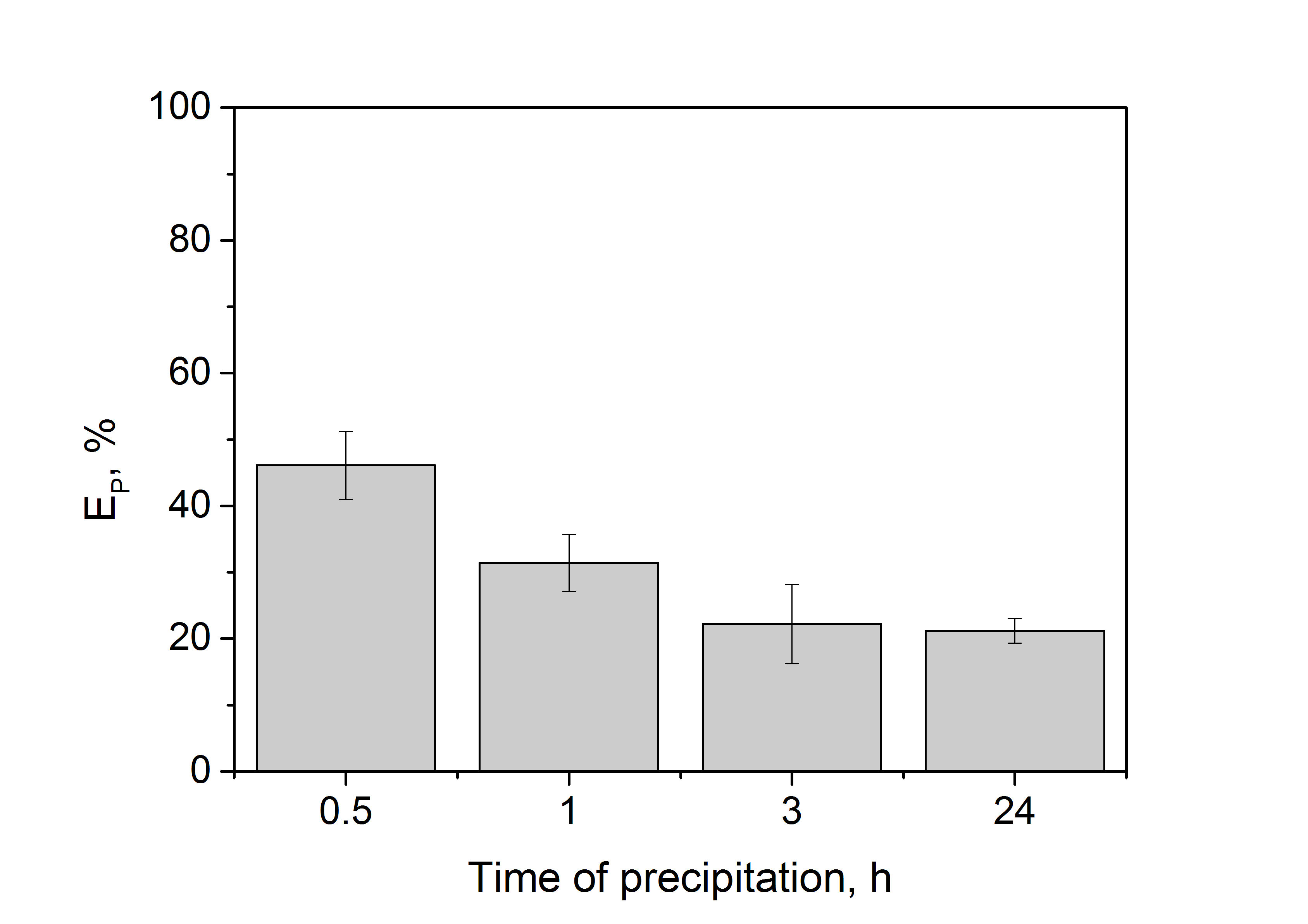 |
| --- | --- |
| **(a)** | **(b)** |

**Fig. A1** (a) UV-vis spectra of solution after Pd precipitation and (b) Pd(II) precipitation yield

Atomic force microscope (AMF) and transmission electron microscopy (TEM) images of 1% PGM@TiO_2_ and particle size distribution after precipitation are presented in Figs. A2 and A3. The precipitation of PGM@TiO_2_ shown in the images was carried out without pH adjustment during the reaction.

| 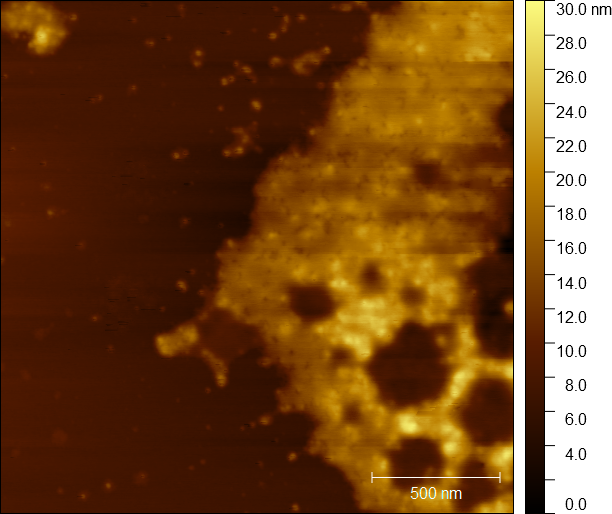 | 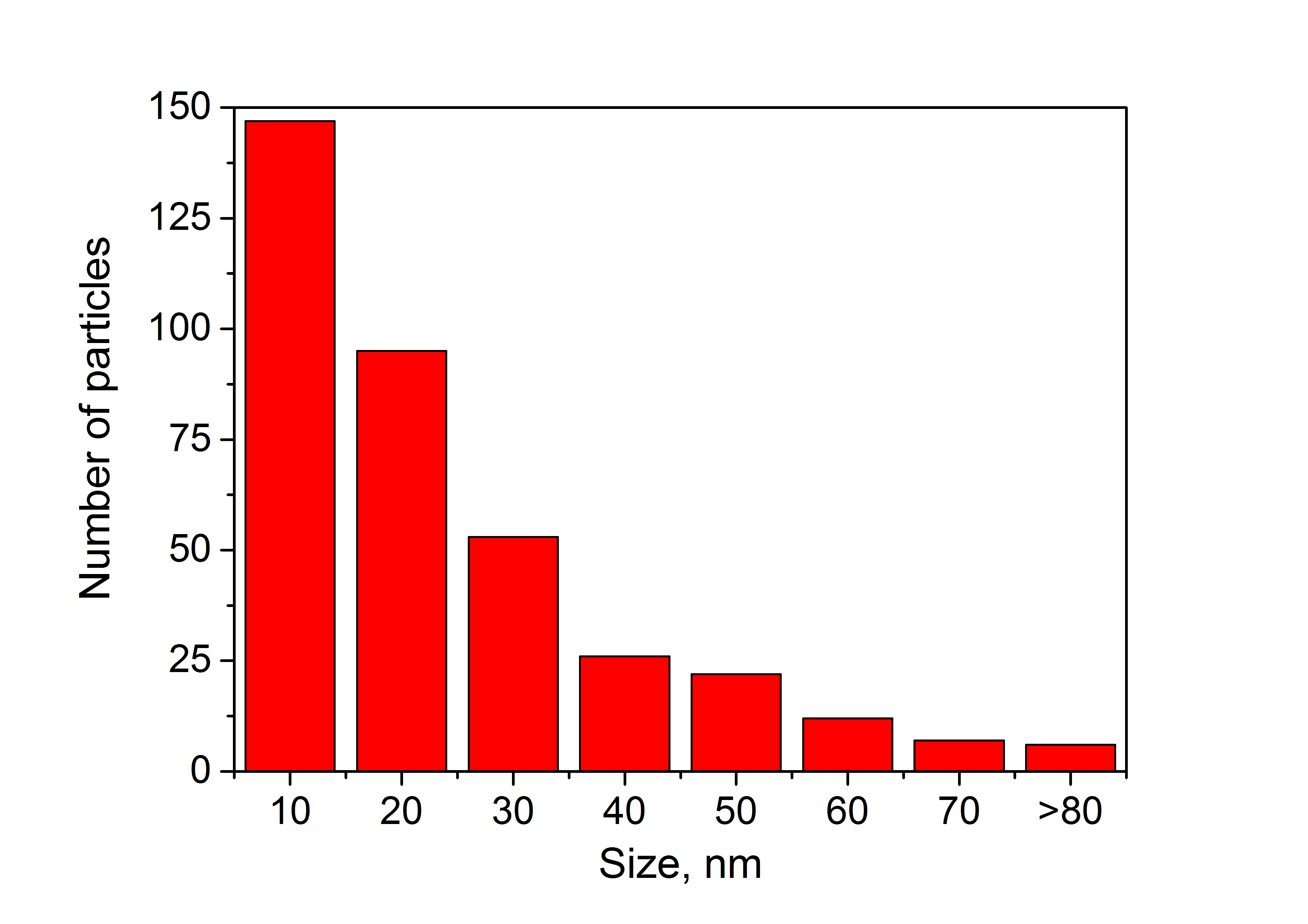 | |
| --- | --- | --- |
| **(a)** | |  |
| 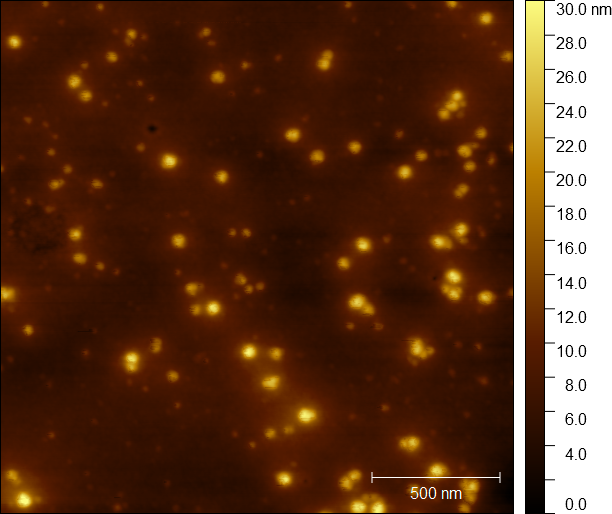 | 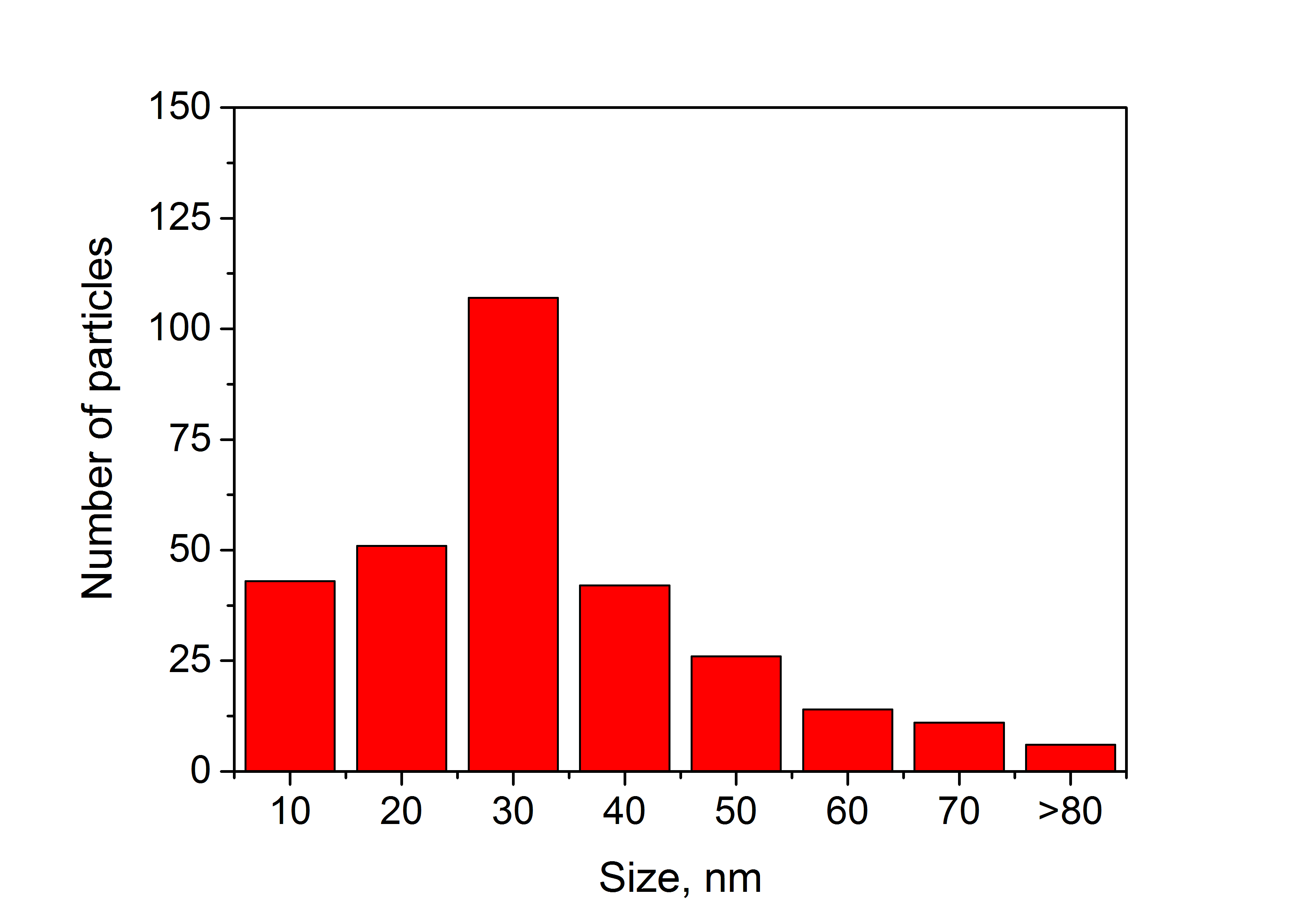 | |
| **(b)** | |  |
| 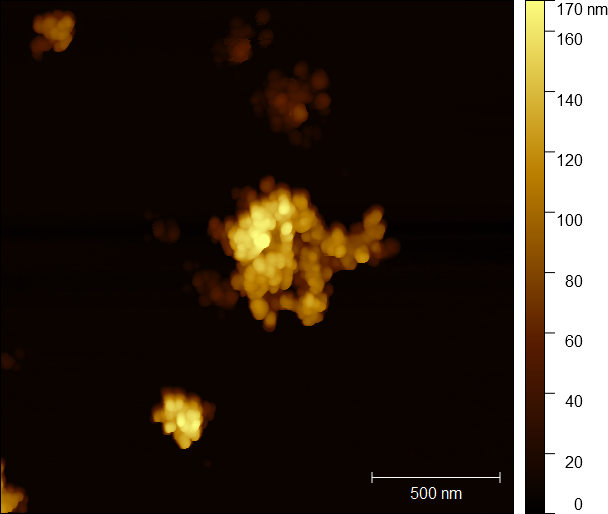 | 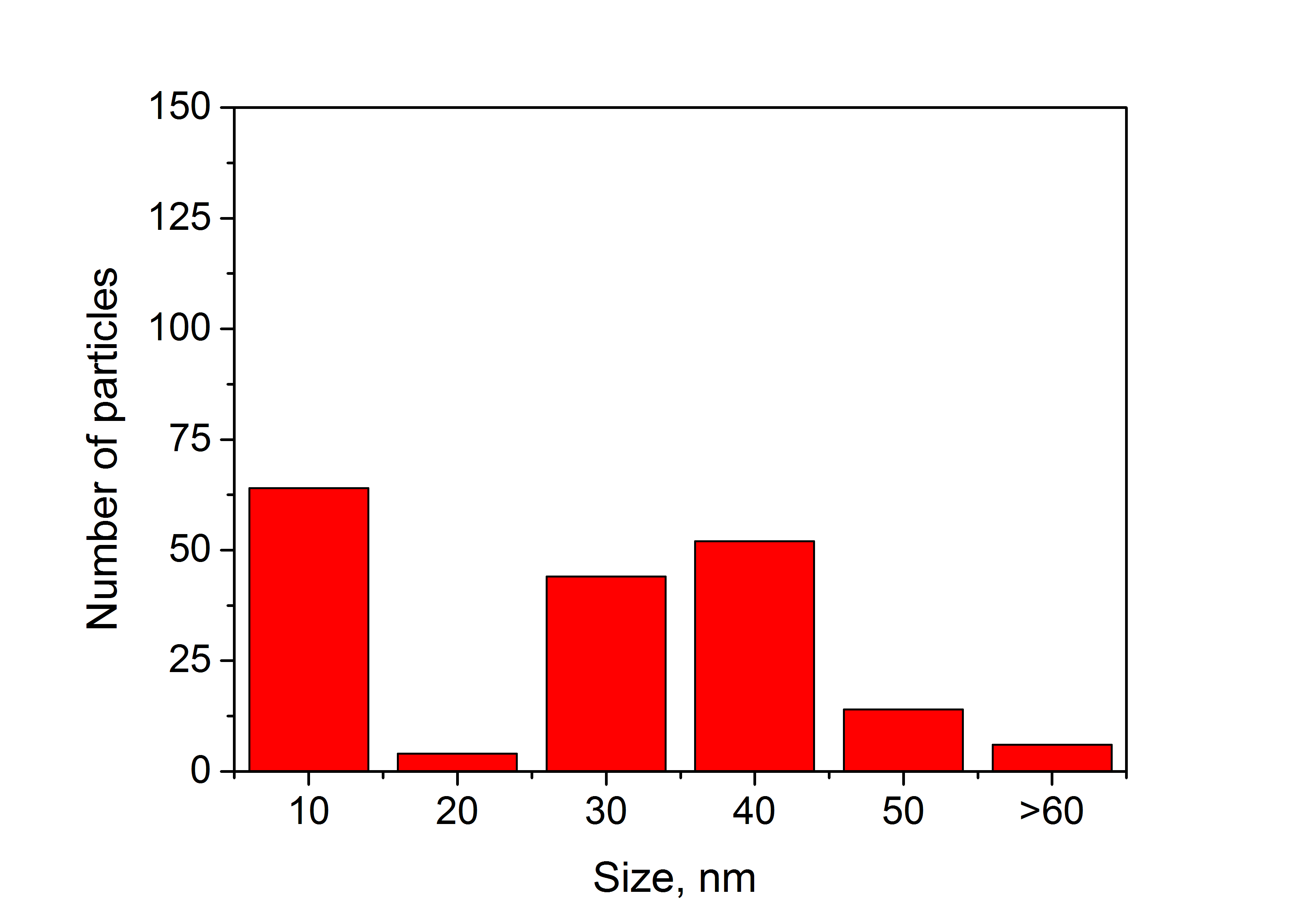 | |
| **(c)** | |  |
| 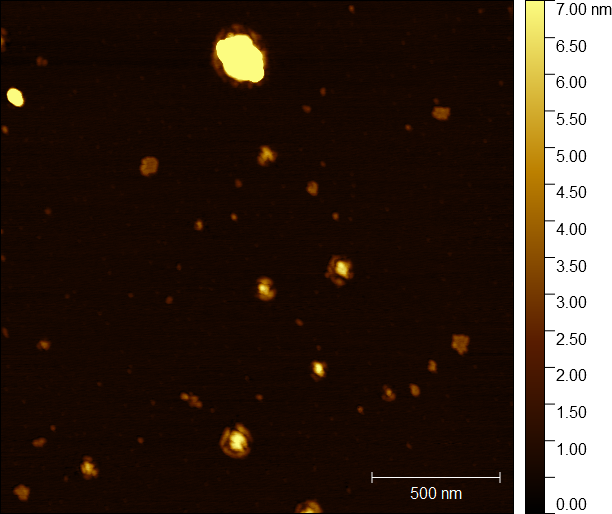 | 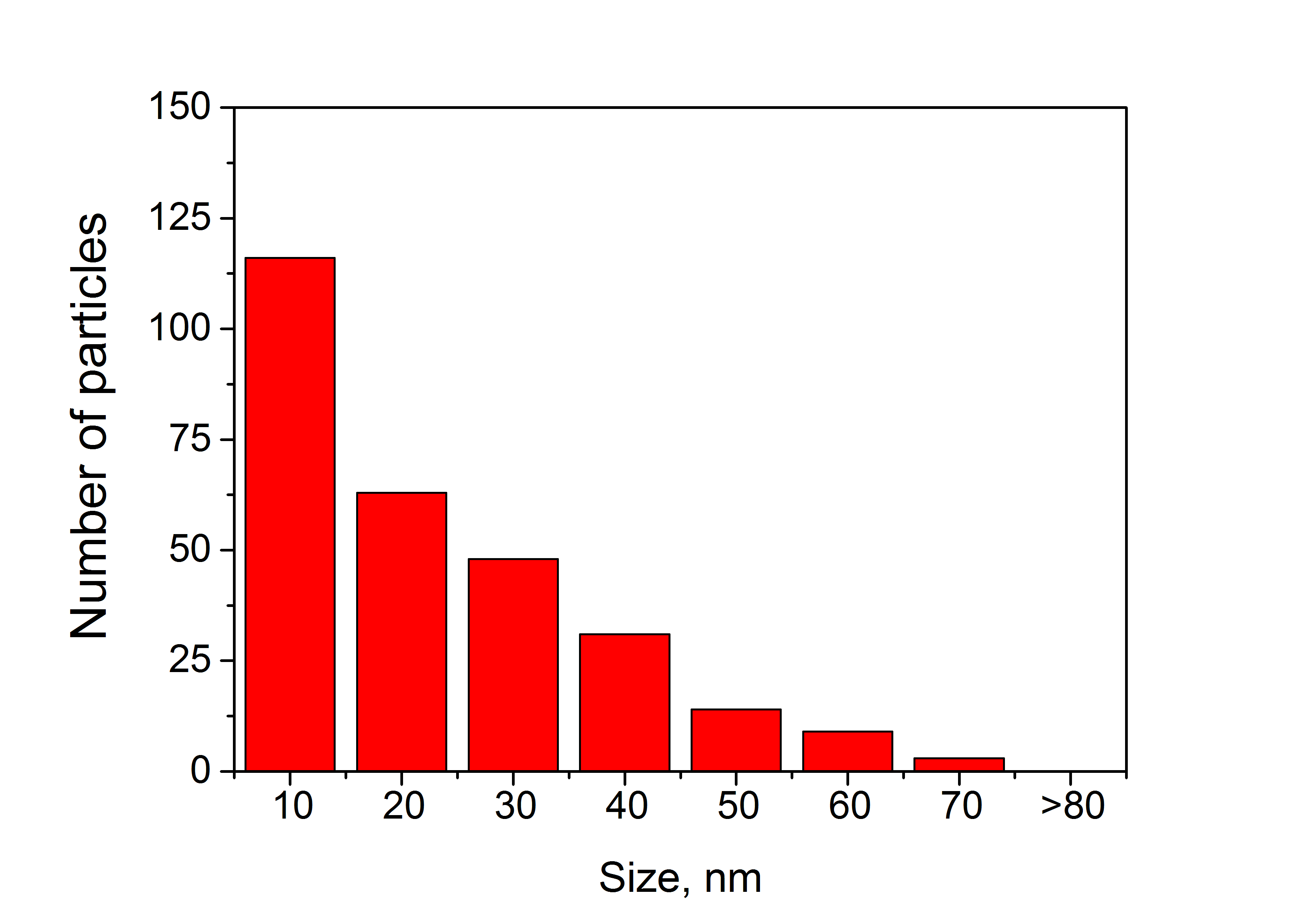 | |
| **(d)** | |  |

**Fig. A2** Atomic Force Microscopy (AFM) images and particle size distribution of (a) TiO_2_, (b) 1% Pt@TiO_2_, (c) 1% Pd@TiO_2_ and (d) 1% Rh@TiO_2_ prepared with pH adjustment

| **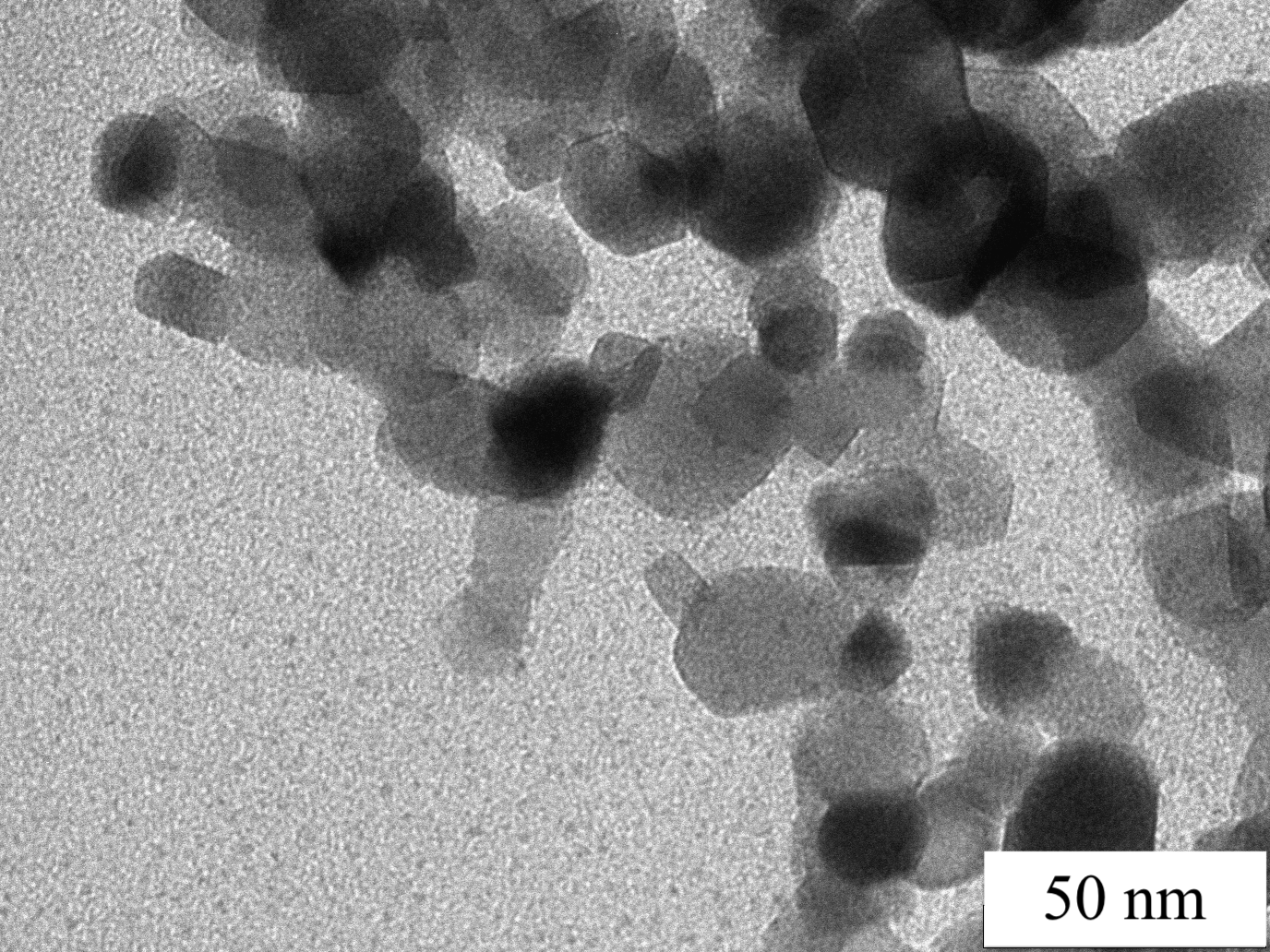**  **(a) 1% Pt@TiO_2_** | **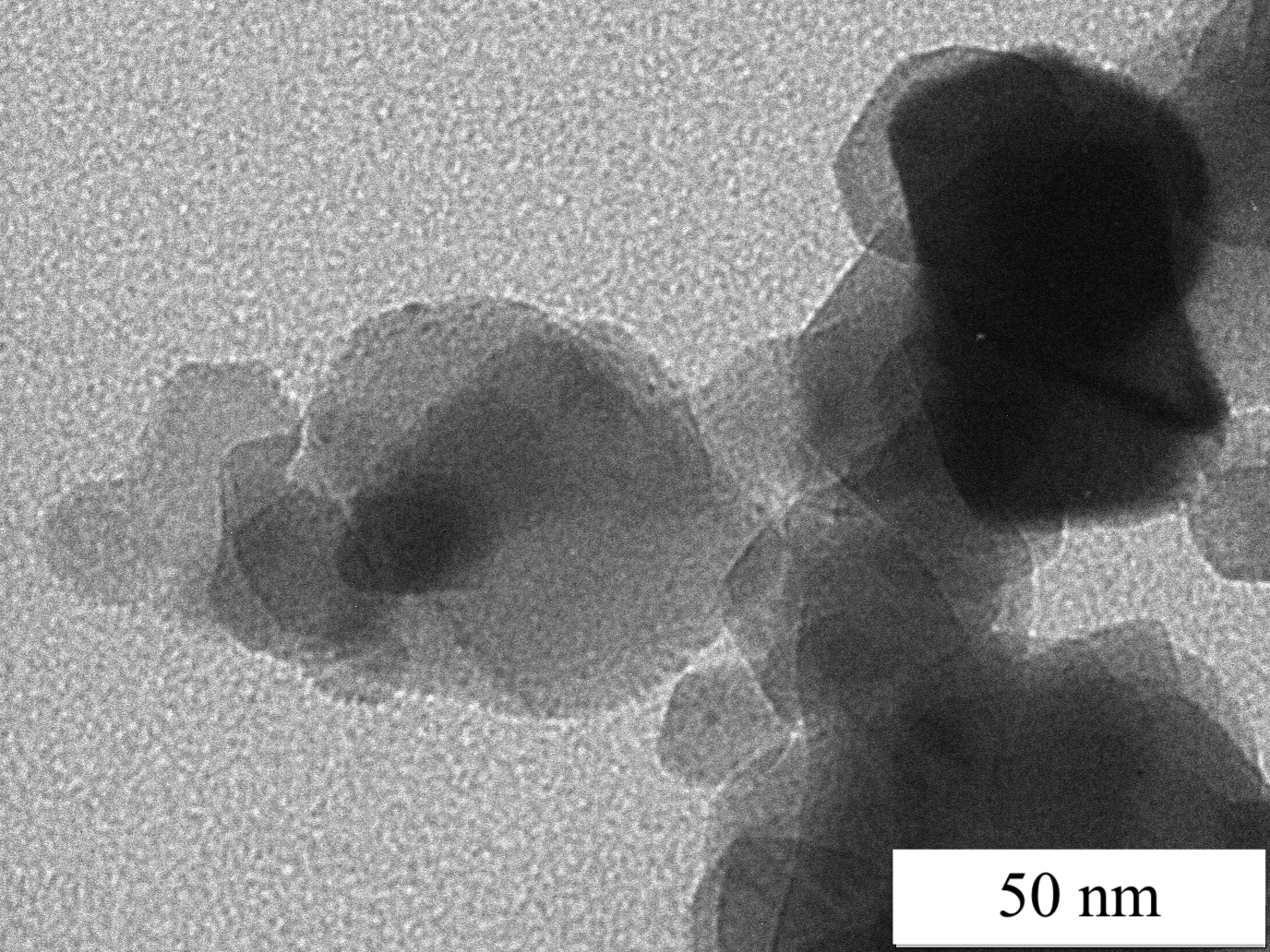**  **(b) 1% Pd@TiO_2_** | **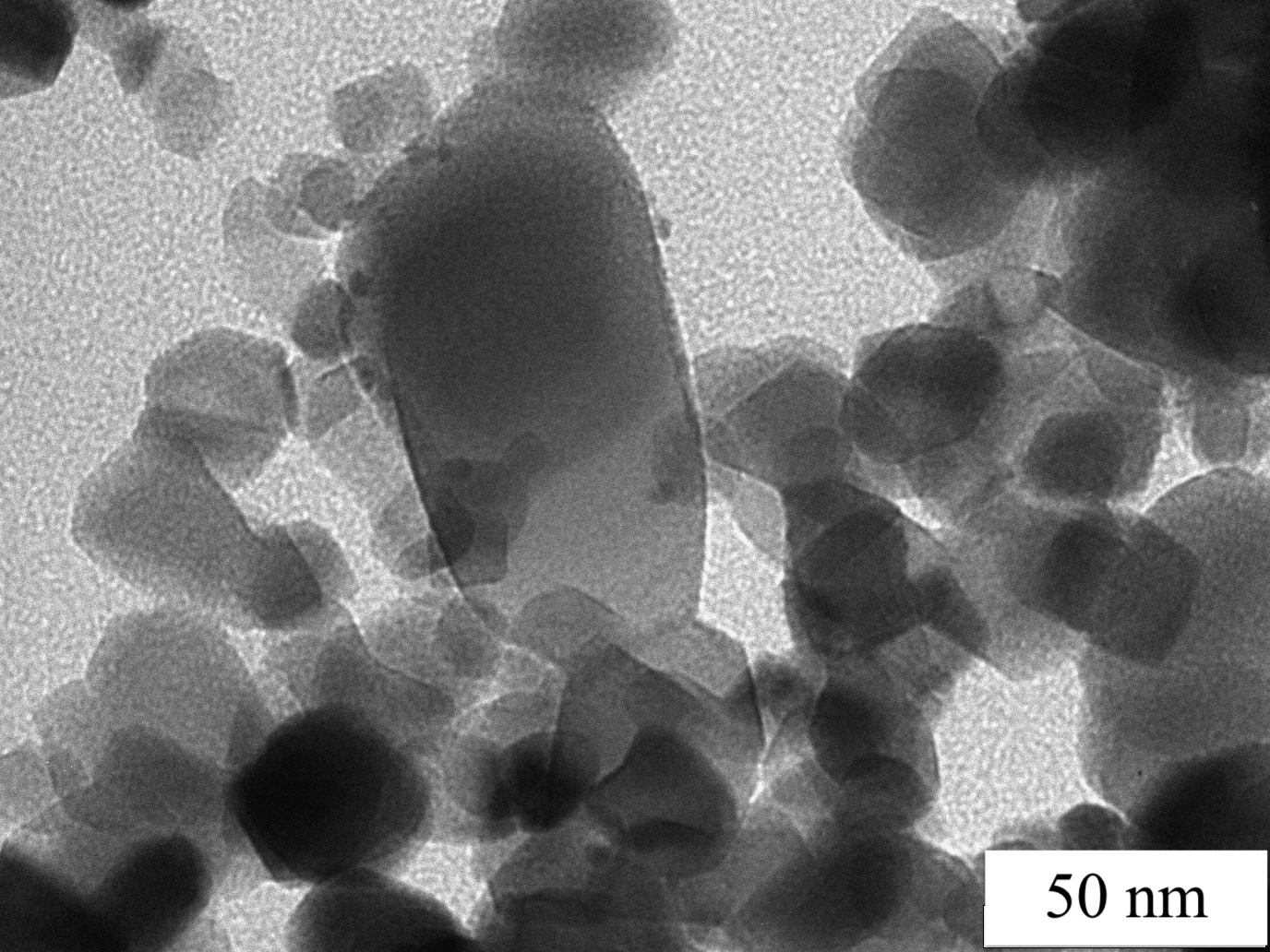**  **(c) 1% Rh@TiO_2_** |
| --- | --- | --- |

**Fig. A3** TEM images of samples after reduction without pH adjustment containing 1 wt.% PGM

Changes in 4-NP concentration and conversion over time using 1% PGM@TiO_2_ showed the highest catalytic activity of Pd NPs over Rh and Pt, which is presented in Fig. A4.





**Fig. A4** Changes in 4-NP concentration (full symbols) and conversion (empty symbols) over time in the presence of 1% PGM@TiO_2._ Reaction conditions: 6 mg catalyst, 30 min reaction time, ambient temperature, reaction medium pH 11

The results shown in Figs. A5-A8 refer to the PGM nanoparticles obtained from the real solution after hydrometallurgical treatment of spent automotive converters.


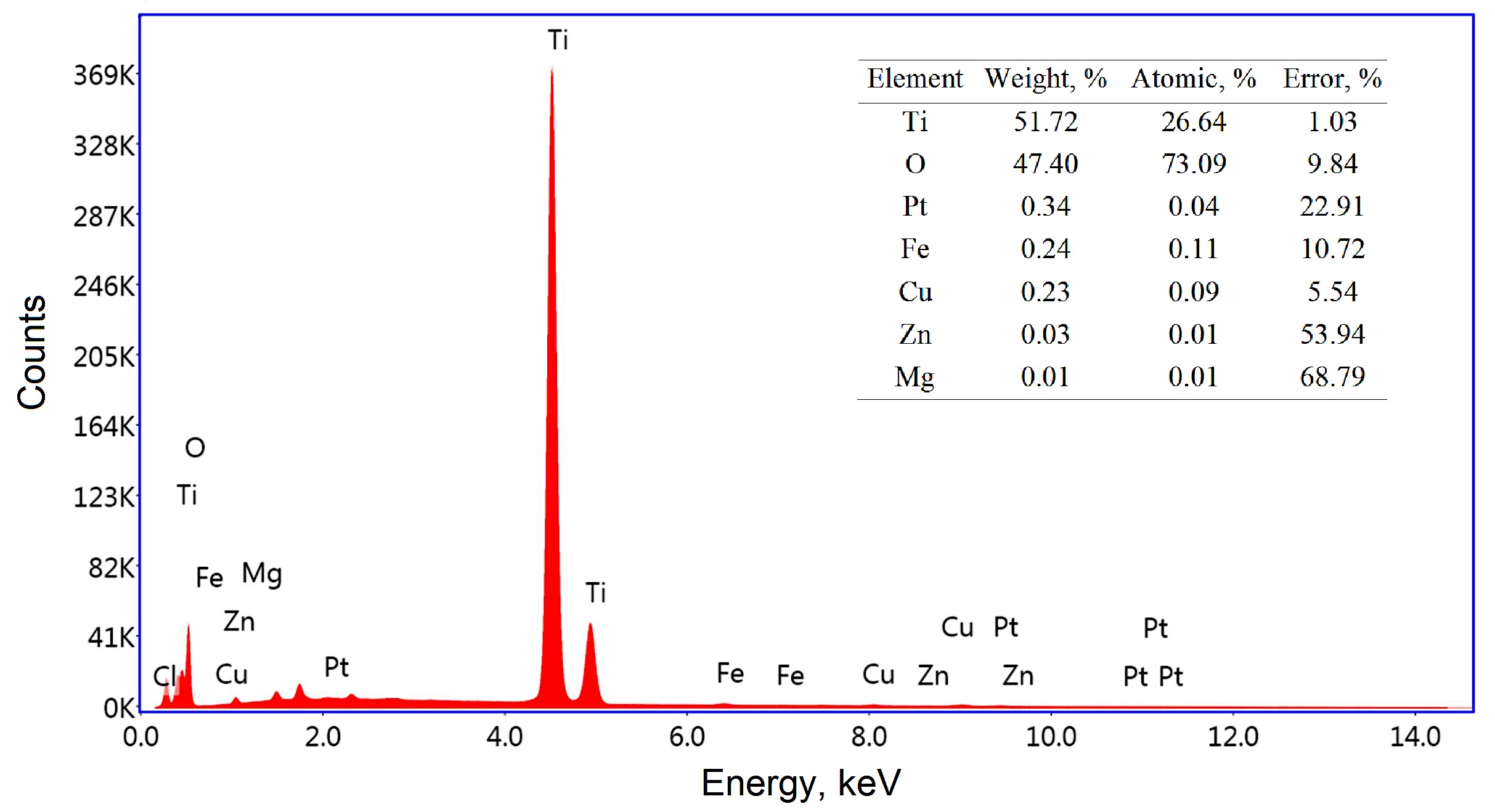


**Fig. A5** SEM-EDS spectrum of Pt@TiO_2_ precipitated from the real leach solution

| 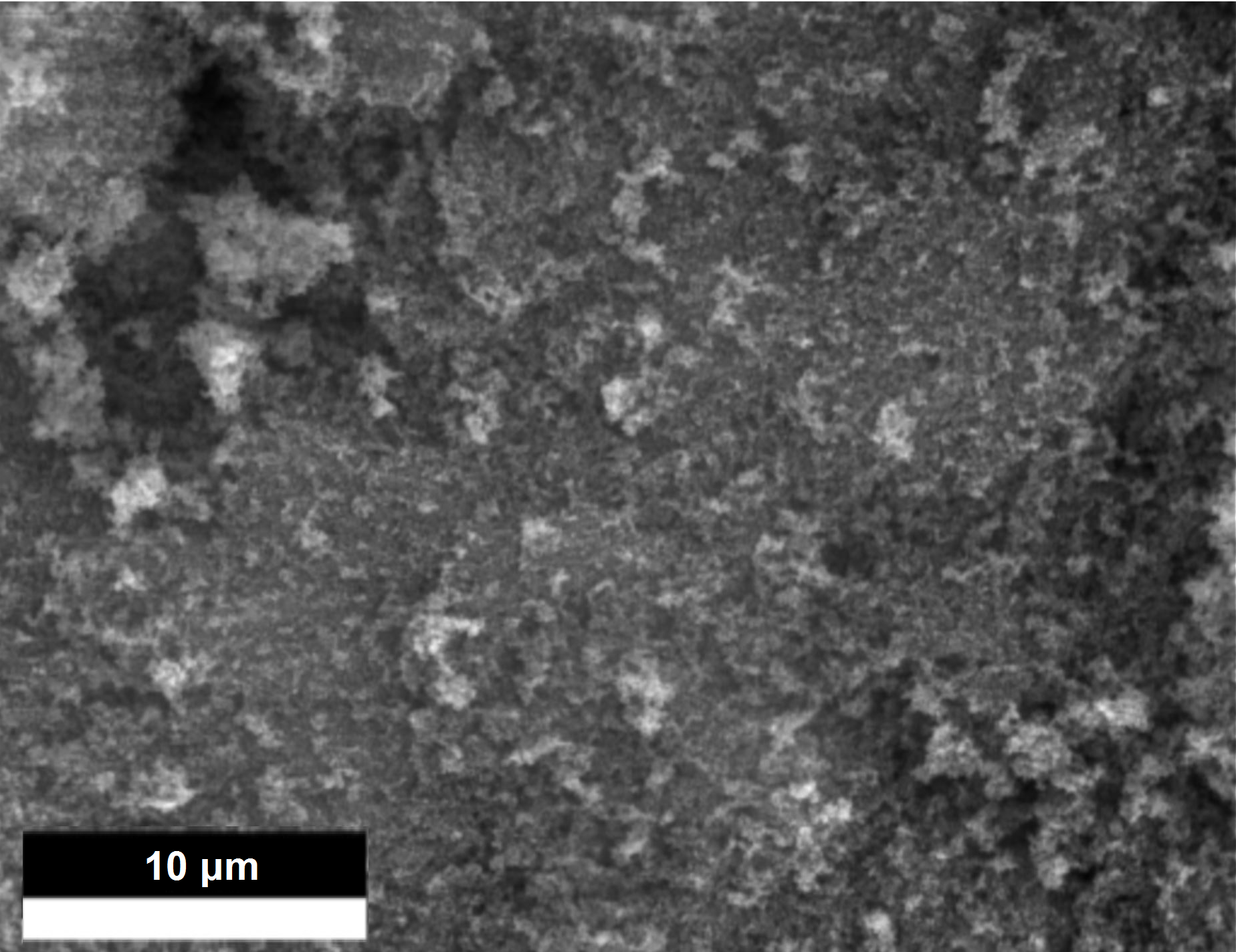 | 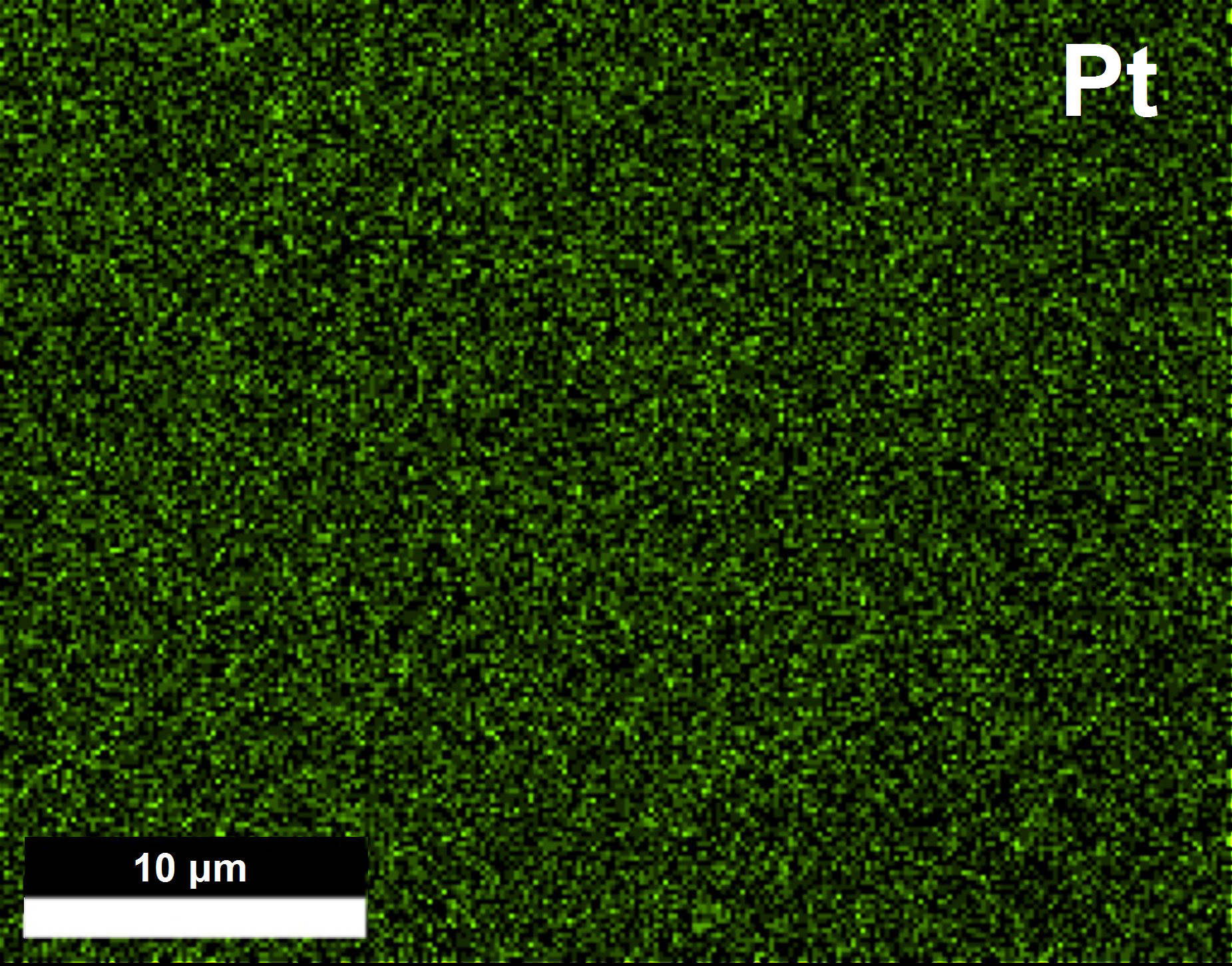 | 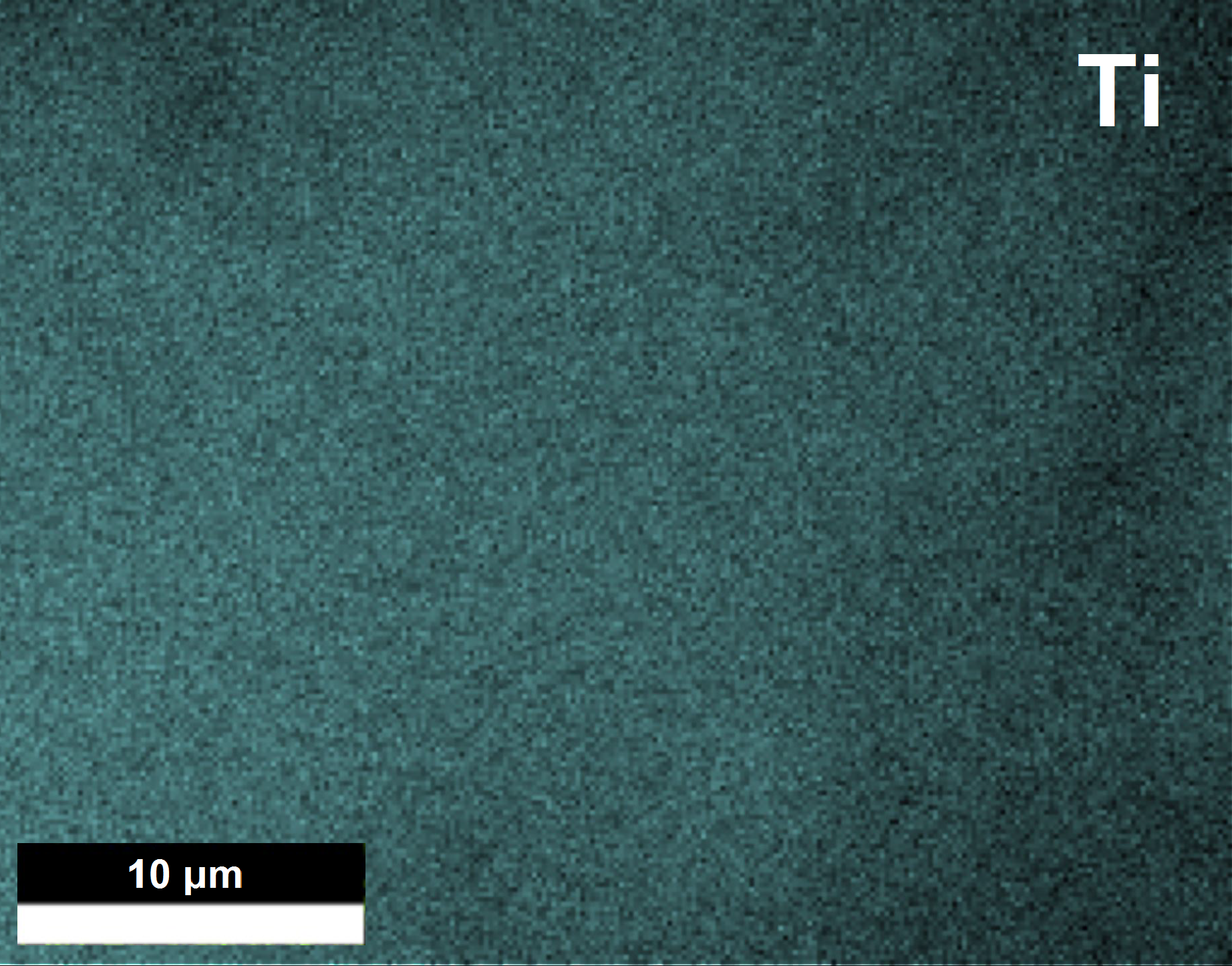 |
| --- | --- | --- |

**Fig. A6** SEM images of sample after reduction from the real leach solution with pH adjustment

| **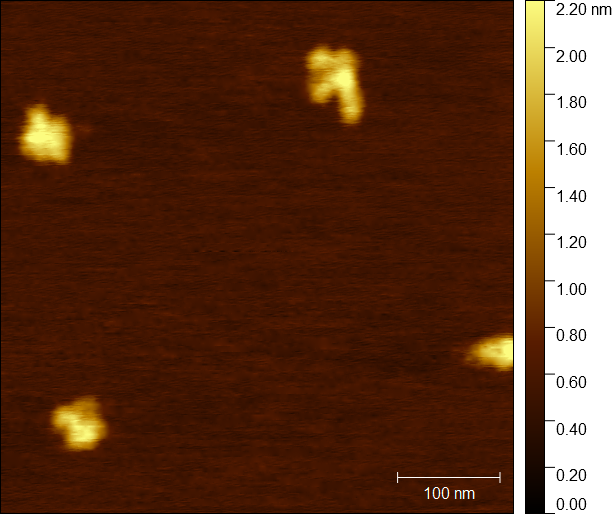** | **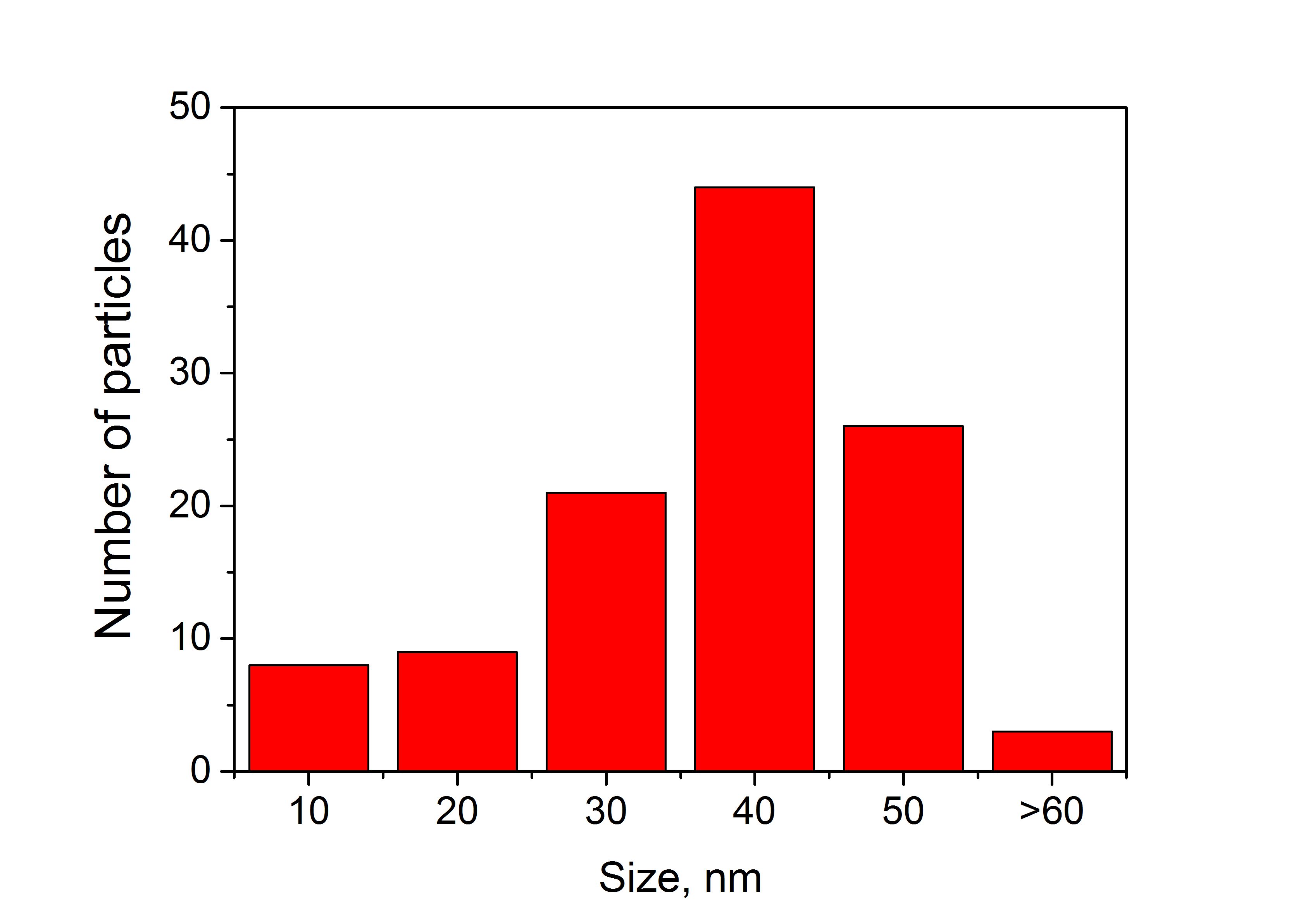** |
| --- | --- |
| **(a)** | **(b)** |

**Fig. A7** AFM analysis of Pt@TiO_2_ precipitated from the real leach solution: (a) AFM image and (b) particle size distribution

| **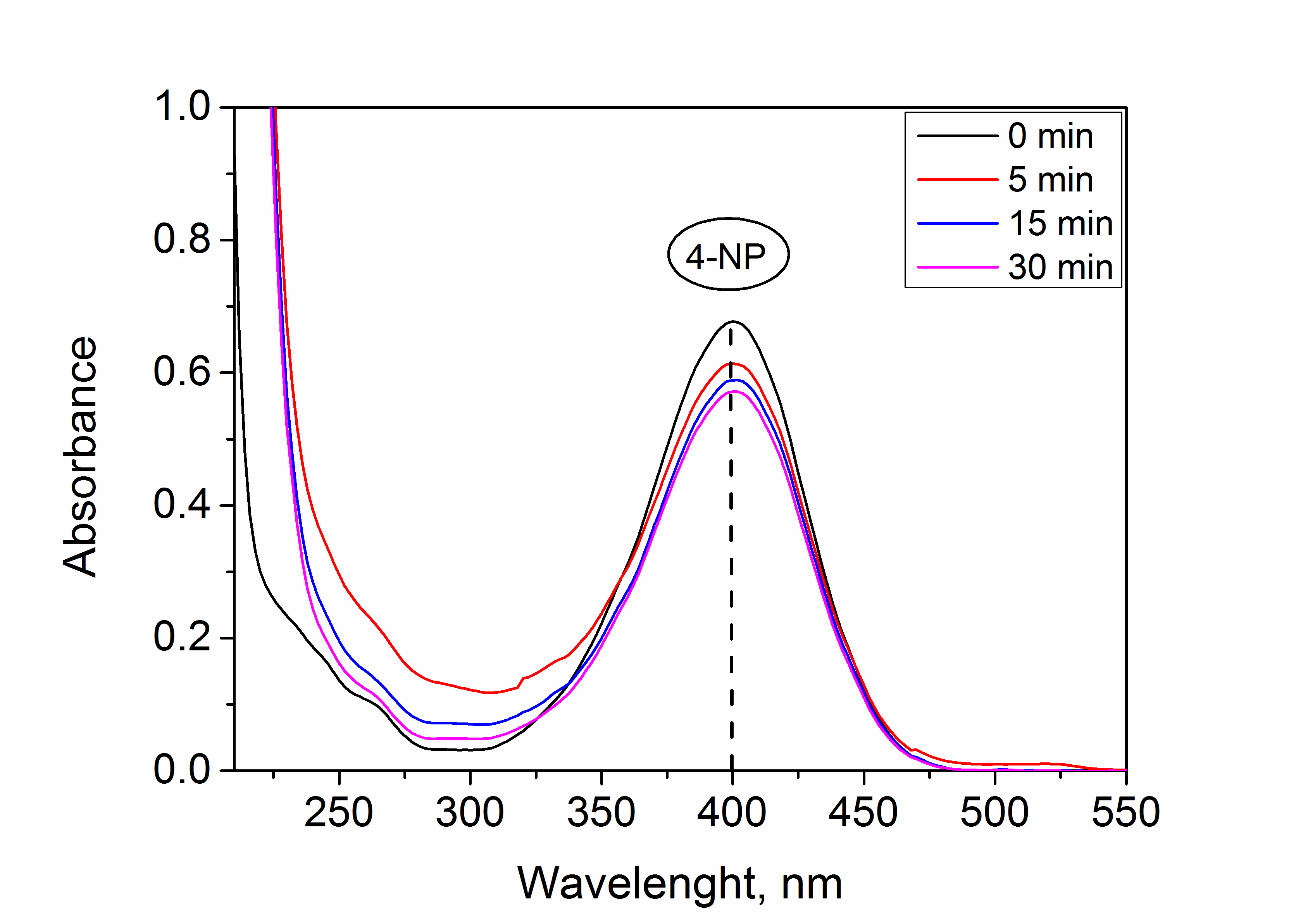** | **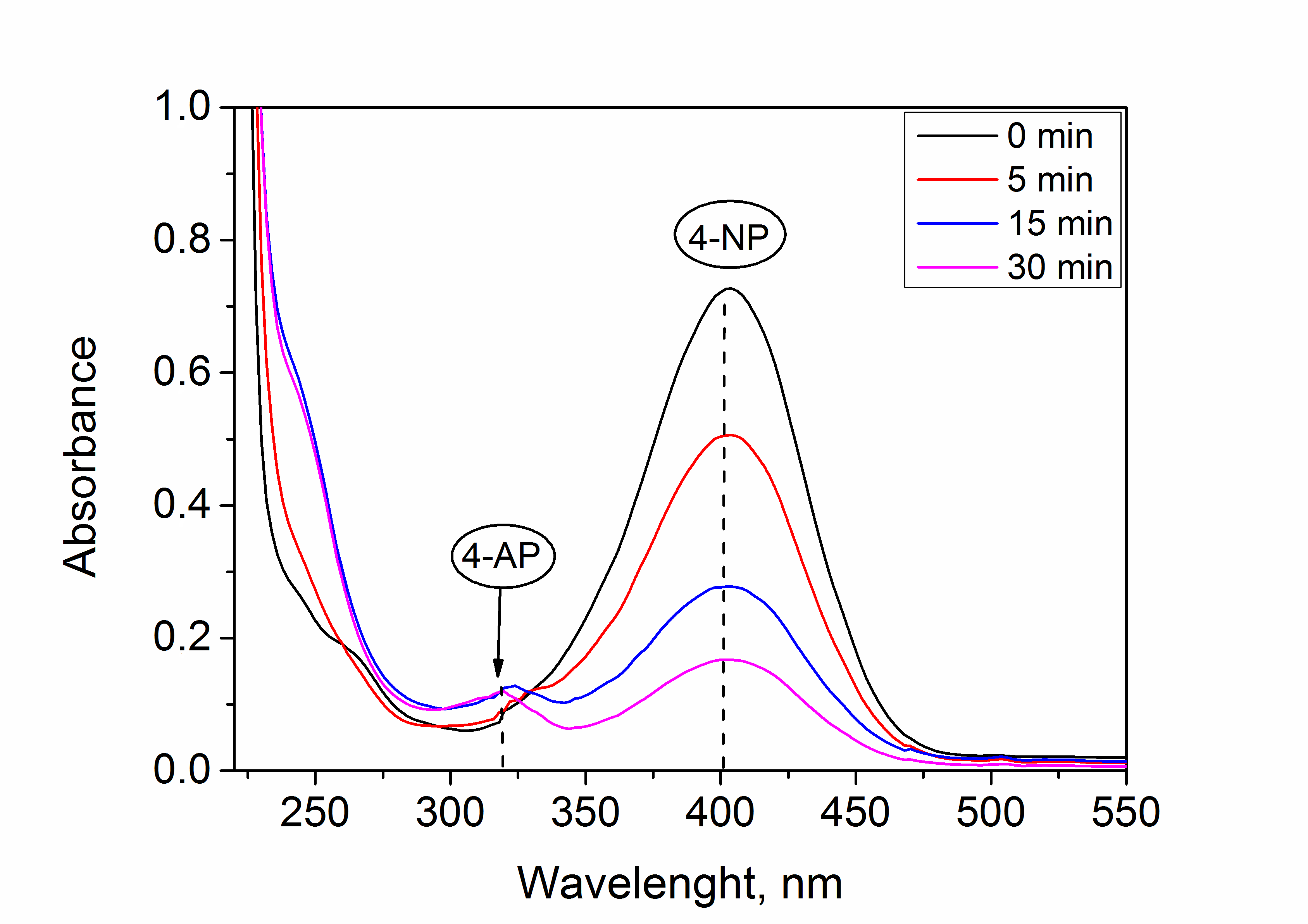** |
| --- | --- |
| **(a)** | **(b)** |

**Fig. A8** UV-vis spectra of the reaction medium at different times in the presence of 1% Pt@TiO_2_: (a) at pH 11 and (b) at pH 14. Reaction conditions: 6 mg catalyst, 30 min reaction time, ambient temperature
